# Engineered heart-liver axis nanoregulators synergize autophagy activation and systemic metabolic reprogramming against atherosclerosis

**DOI:** 10.1126/sciadv.aee3352

**Published:** 2026-07-31

**Authors:** Yiyong Tang, Jiadi Liu, Liya Tian, Bowen Shen, Tairen Zhou, Shen Zhao, Qianqian Gao, Mingda Du, Yuanchen Sun, Xiao Sun, Houren Zhou, Weihai Xu, Jun Wu

**Affiliations:** ^1^ Cardiovascular Ultrasound Department, The Second Affiliated Hospital of Dalian Medical University, Dalian, China.; ^2^ Shandong Cancer Hospital and Institute, School of Chemistry and Pharmaceutical Engineering, Medical Science and Technology Innovation Center, Shandong First Medical University and Shandong Academy of Medical Sciences, Jinan, China.; ^3^ School of Pharmaceutical Engineering, Shenyang Pharmaceutical University, Shenyang, China.; ^4^ Department of Neurology, State Key Laboratory of Complex Severe and Rare Diseases, Peking Union Medical College Hospital, Chinese Academy of Medical Sciences and Peking Union Medical College, Beijing, China.

## Abstract

Atherosclerosis (AS) is the most important pathological basis for cardiovascular diseases worldwide. However, the current mainstream therapeutic strategies for AS only target isolated pathological links, ignoring hepatic steatosis as a related risk factor for AS, which leads to limited effectiveness in controlling the overall course of atherosclerosis. Herein, a smart nanoplatform that can synergistically regulate AS and hepatic steatosis was first reported, which featured sulfide iron nanosheets as the core, grafted with bovine myeloid antimicrobial peptide 27 (BMAP-27), skillfully coated with an erythrocyte-macrophage hybrid biomimetic membrane. First, this nanoplatform is capable of targeting atherosclerotic plaques. When combined with low-intensity focused ultrasound, the nanoplatform markedly enhanced local drug accumulation. The localized temperature rise generated by near-infrared laser irradiation acted to open TRPV1 channels, facilitating Ca^2+^ entry. This increase in cytosolic Ca^2+^ activated autophagy in foam cells, upregulated ABCA1-mediated cholesterol efflux, and reduced oxidized low-density lipoprotein accumulation. Meanwhile, FPRM efficiently scavenged reactive oxygen species (ROS) within atherosclerotic plaques and synergized with BMAP-27 to suppress AS-related inflammation. Intriguingly, the classic hepatic accumulation-metabolism pathway of FPRM enabled continuous ROS elimination in the liver, effectively alleviating hepatic steatosis and lowering plasma triglyceride levels, thereby achieving metabolic reprogramming and ultimately inhibiting the progression of atherosclerosis.

## Teaser

Engineered heart-liver axis nanoregulators enable autophagy and systemic metabolic reprogramming to treat atherosclerosis.

## INTRODUCTION

Atherosclerosis (AS) is a chronic vascular disease defined by endothelial oxidative stress, dysregulated lipid metabolism, and persistent chronic inflammation. It constitutes a fundamental pathological basis for a range of fatal conditions, including coronary heart disease and aortic dissection, and remains a leading cause of mortality worldwide (*1*–*3*). Chronic and unremitting inflammation is one of the core factors driving the occurrence and progression of AS (*4*, *5*). In the atherosclerotic lesion area, activated macrophages produce excessive reactive oxygen species (ROS). This not only exacerbate oxidative damage but also promote the release of inflammatory cytokines, thereby forming and worsening the local inflammatory microenvironment (*6*–*8*). Beyond inflammation and oxidative stress, the early pathological features of AS also include the abnormal accumulation of cholesterol in macrophages and vascular smooth muscle cells (VSMCs), culminating in foam cell formation (*9*–*11*). Moreover, there is a close pathophysiological connection between hepatic steatosis and atherosclerosis (*12*, *13*). A large amount of evidence indicates that hepatic steatosis is not only an independent risk factor for AS but also significantly promotes its occurrence and development (*14*, *15*). As the pathological basis of cardiovascular disease, AS plays a key bridging role between hepatic steatosis and increased cardiovascular event risk. Hepatic steatosis and AS form a mutually reinforcing vicious cycle through shared pathways such as systemic inflammation, oxidative stress, and lipid metabolism disorders. Therefore, intervening in AS while ignoring its liver-driven factors, or improving hepatic steatosis while neglecting its vascular complications, is unlikely to achieve ideal long-term efficacy. Developing a combined “cardio-hepatic co-treatment” strategy aims to simultaneously block this pathological network by actively alleviating hepatic steatosis while intervening in AS, synergistically improving lipid metabolism and reducing inflammatory burden at the systemic level. This is of crucial importance for enhancing the overall efficacy of cardiometabolic diseases and fundamentally reducing cardiovascular risk. However, currently, clinical drugs used for treating AS (e.g., lipid-lowering drugs and antithrombotic drugs) mostly address isolated pathological processes. There is a lack of comprehensive treatment strategies that can simultaneously intervene in multiple progressive mechanisms of the disease.

Recent studies have found that the transient receptor potential vanilloid subtype 1 (TRPV1) channel, a heat-sensitive cation channel, can be stimulated by agonists such as capsaicin. It opens at approximately 42°C, promoting Ca^2+^ influx and inducing protective autophagy (*16*–*18*). This autophagic process can encapsulate cytoplasmic oxidized low-density lipoprotein (oxLDL) into double-membraned vesicles, which then fuse with lysosomes (*19*, *20*). In lysosomes, acid lipase hydrolyzes oxLDL into free cholesterol, which is mainly excreted from cells through the mediation of ATP-binding cassette transporter A1 (ABCA1), thereby reducing lipid deposition (*21*, *22*). Based on this mechanism, the activation of TRPV1 is considered a promising target for the treatment of AS. However, capsaicin, as a classic agonist, has problems of systemic toxicity and uncontrollable diffusion, which easily cause off-target effects and severely limit its clinical application (*23*, *24*). Therefore, achieving precise spatiotemporal regulation of TRPV1 signaling in vivo is particularly crucial.

**Fig. 1.**
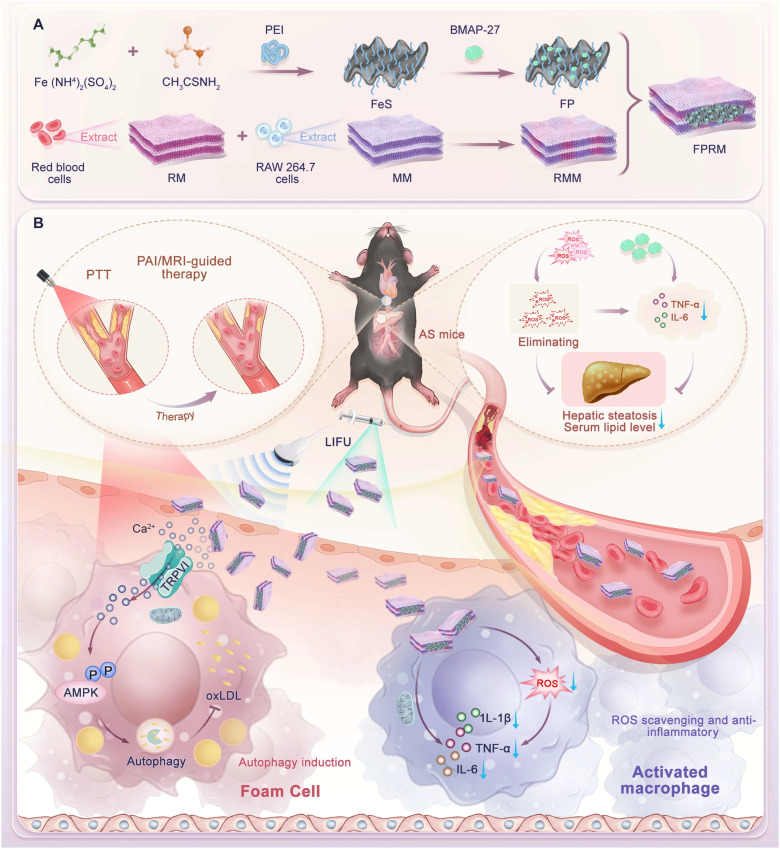
Schematic illustration of the engineered heart-liver axis nanoregulators for atherosclerosis therapy via synergistic autophagy activation and systemic metabolic reprogramming. (**A**) The nanoplatform was constructed by grafting the anti-inflammatory peptide BMAP-27 onto FeS nanosheets and coating them with the RMM for active targeting to atherosclerotic plaques. (**B**) Upon arrival at the plaque site and assisted by LIFU for enhanced penetration, the nanoplatform generated mild hyperthermia under NIR laser irradiation. This local temperature rise activated the TRPV1 channel, triggering Ca^2+^ influx, inducing protective autophagy, and promoting ABCA1-mediated cholesterol efflux to reduce lipid accumulation in foam cells. Concurrently, the FeS nanosheets efficiently scavenged reactive oxygen species (ROS) and, together with BMAP-27, synergistically alleviated plaque inflammation. Furthermore, the nanoplatform enabled PA and MR dual-modal imaging for visualizing atherosclerotic lesions. By leveraging its intrinsic liver-accumulation property, the nanoplatform simultaneously scavenged intrahepatic ROS, suppressed inflammation, ameliorated hepatic steatosis and systemic dyslipidemia, ultimately achieved systemic metabolic reprogramming, and thereby inhibited atherosclerotic progression.

Based on the above multiple factors, we have constructed an intelligent nanoplatform capable of synergistically regulating AS and hepatic steatosis (Fig. 1). This nanoplatform was constructed by grafting bovine myeloid antimicrobial peptide 27 (BMAP-27) with significant anti-inflammatory activity onto FeS nanosheets, followed by coating with the red blood cell-macrophage hybrid biomimetic membrane (RMM) to achieve specific targeting of atherosclerotic plaques. Under the cavitation effect induced by low-intensity focused ultrasound (LIFU), FPRM (FeS/BMAP-27@RMM) could precisely accumulate in the plaque area, transiently enhance cell membrane permeability, and significantly improve the uptake and retention of drugs at the lesion site. This two-dimensional (2D) nanoplatform exhibited excellent photothermal conversion efficiency (PCE, η = 30%). Under near-infrared (NIR) irradiation, local temperature elevation activated the TRPV1 channel, promoted Ca^2+^ influx, thereby activating cellular autophagy, upregulating ABCA1-mediated cholesterol efflux, and reducing oxLDL accumulation. Meanwhile, FeS nanosheets scavenged excessive ROS through strong antioxidant reactions and synergized with BMAP-27 to exert anti-inflammatory effects, collectively alleviating the progression of AS. In addition, FPRM possessed excellent photoacoustic (PA) and magnetic resonance (MR) imaging capabilities, providing visual support for the precise spatiotemporal regulation of the TRPV1 pathway in vivo. Unexpectedly, the most classic liver accumulation and metabolism pathway of the nanoplatform had also become an advantage. In the liver, FeS nanosheets effectively scavenged ROS and synergize with BMAP-27 to reduce inflammatory responses, thereby effectively alleviating hepatic steatosis, improving lipid metabolism, lowering plasma triglyceride levels, and ultimately achieving systemic metabolic reprogramming. To sum up, the multifunctional FPRM nanoplatform realized the synergistic regulation of metabolic disease-related systems by simultaneously inhibiting the development of atherosclerotic plaques and alleviating hepatic steatosis, constructing an integrated “heart-liver synergistic treatment” system, which provided a new approach for improving the overall efficacy of AS (Fig. 1).

## RESULTS

### Synthesis and characterization of FPRM

**Fig. 2.**
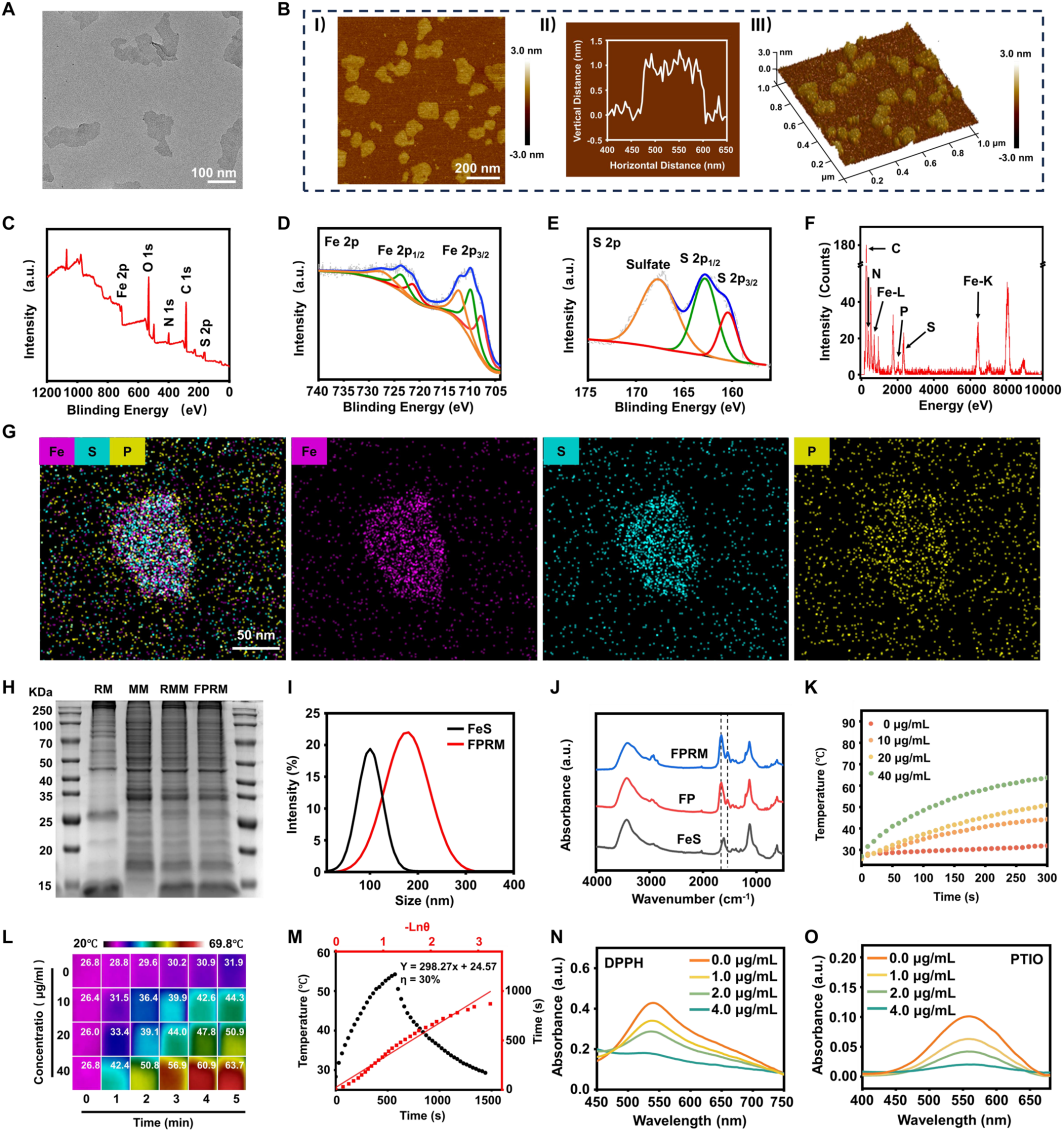
Fabrication and characterizations of FPRM. (**A**) TEM image of FeS. Scale bar, 100 nm. (**B**) (I and III) AFM images of FeS nanosheets and (II) its corresponding height diagram. Scale bar, 200 nm. (**C**) XPS survey spectrum of FeS nanosheets. (**D**) Fe2p XPS spectra of FeS. (**E**) S2p XPS spectra of FeS. (**F**) The EDS analysis of FPRM nanosheets. (**G**) Elemental mapping analysis of FPRM. Scale bar, 50 nm. (**H**) SDS-PAGE analysis of membrane protein conservation during the preparation of FPRM. (**I**) DLS analysis of FeS and FPRM. (**J**) FTIR of FeS, FP and FPRM. (**K**) The photothermal heating profiles of FPRM at various concentrations were recorded upon exposure to an 808 nm laser at 1.0 W/cm^2^ for 5 minutes. (**L**) The NIR photothermal imaging of FPRM with increasing irradiation time and concentration. (**M**) Calculated PCE of FPRM. (**N**) DPPH•-eliminating capability of the FPRM monitored through UV-vis spectrum. (**O**) PTIO•-eliminating capability of the FPRM monitored through UV-vis spectrum.

Initially, FeS was prepared in a high-pressure reactor via the solvothermal approach. The synthesis of FPRM nanosheets was accomplished through a three-step procedure (Fig. 1). Transmission electron microscopy (TEM) results revealed that FeS possessed an ultrathin morphology (Fig. 2A). The thickness of the FeS nanosheets was validated by atomic force microscopy (AFM), with measurements determined to be below 2 nm (Fig. 2B). The x-ray photoelectron spectroscopy (XPS) survey spectrum of the nanosheets detected the characteristic peaks of iron (Fe), sulfur (S), oxygen (O), carbon (C), and nitrogen (N) elements (Fig. 2C). Moreover, the analysis of the Fe 2p and S 2p detail spectrum showed the presence of Fe^2+^ and S^2−^ (Fig. 2, D and E). Meanwhile, the x-ray diffraction (XRD) peaks of the synthesized nanosheets matched the characteristic pattern of FeS (fig. S1). Furthermore, investigations were conducted to assess the modification of cell membrane on the surface of nanosheets. The TEM image of FPRM revealed that FPRM still maintained sheet-like structure (fig. S2). In addition, elemental mapping analysis (Fig. 2G) and energy-dispersive spectrometry (EDS) analysis (Fig. 2F) showed the presence of phosphorus (P) element on the nanosheets, which was a key component of cell membrane.

As the preparation process progressed, it was found that the size gradually increased from FeS to FPRM (Fig. 2I). Additionally, the potential exhibited a continuous downward trend, especially with the Zeta potential of FPRM shifting to a negative value (fig. S3). This notable change was directly attributed to the inherent abundance of negative charges in the cell membrane itself. Membrane protein retention results showed that the protein bands of the hybridized membrane and FPRM were similar to those of the erythrocyte membrane (RM) and the membrane of RAW 264.7 cells (MM), indicating that the membrane protein remained stable after undergoing sonication, centrifugation, and the working temperature associated with the membrane extraction process (Fig. 2H). During the coating process, membrane protein can be effectively transferred onto the FPRM along with the lipid membrane, ensuring that subsequent cellular membrane functions were maintained. In Fourier transform infrared spectroscopy (FTIR), a characteristic peak between 3000 and 2700 cm^−1^ can be classified as C-H stretching vibration peak, indicating that FeS nanosheets were synthesized under the driving force of polyethyleneimine (PEI). FPRM showed amide vibration peak (VC=O, VC-N, 1646 cm^−1^, 1554 cm^−1^), which were indicative of the successful cell membrane coating (Fig. 2J and fig. S4). In the Coomassie brilliant blue staining assay, the presence of a protein band in the FP group that aligned with BMAP-27 suggested the successful incorporation of the peptide (fig. S5). Based on optical density measurements, the grafted BMAP-27 content in FP was determined to be 1.596%. Collectively, these findings supported the successful synthesis of FPRM.

The absorbance increased with higher concentrations of FeS by UV-vis spectroscopy (fig. S6). This finding indicated that FeS nanosheets possessed strong absorption capability, which provided the possibility for their application in photothermal therapy. After the preparation of FPRM, its photothermal performance was tested. Following irradiation with an 808 nm laser (power density: 1.0 W/cm^2^), the photothermal performance of FPRM was assessed across a range of concentrations (0, 10, 20, and 40 μg/ml) (Fig. 2K). Notably, at a concentration of 10 μg/ml, the solution temperature increased to approximately 44°C after 5 min of irradiation, which was within the effective temperature range for mild photothermal therapy. Under this mild photothermal condition, comparative SDS-PAGE analysis showed that the membrane protein profiles of FPRM remained largely unchanged before and after NIR irradiation, indicating good stability of the integrated membrane components (fig. S7). Additionally, we assessed the photothermal effect at varying laser power densities (0.5, 1.0, and 1.5 W/cm^2^) (fig. S8), and the results confirmed the correlation between the thermal effect of FPRM and power density. Furthermore, the thermal imaging data (Fig. 2L) directly verified a significant temperature rise of FPRM, which exhibited a clear concentration-dependent and time-dependent profile under NIR irradiation. Fig. 2M showed that the photothermal conversion efficiency of FPRM reached up to 30%. Over five laser on/off cycles, the peak temperature of FPRM decreased to a certain extent, indicating that the FPRM had a trend of gradually degrading with appropriate photothermal stability (fig. S9).

The excessive production of ROS coupled with sustained oxidative stress initiated cellular and tissue damage, provoked inflammatory cascades, and culminated in a self-amplifying cycle of oxidative damage. The pathogenesis of atherosclerosis (AS) is critically driven by the excessive generation of ROS. Thus, the free radical scavenging ability of FPRM was subsequently determined by several standard assays. In the field of antioxidant activity detection, the 2,2-diphenyl-1-picrylhydrazyl radical (DPPH•) assay is extremely widely used. Initially, this method was utilized to assess the free radical scavenging capacity of FPRM. The results demonstrated that when the Fe^2+^ concentration in the system reached 4 μg/ml, the scavenging rate of DPPH• by FPRM was greater than 60%, indicating that FPRM exhibited a relatively strong free radical scavenging performance (Fig. 2N and fig. S10). Although the DPPH• detection method is commonly used in the evaluation of antioxidant capacity, it has certain limitations and cannot comprehensively and accurately reflect the antioxidant capacity of substances. Therefore, another widely used oxygen-centered radical, 2-phenyl-4,4,5,5-tetramethylimidazoline-1-oxyl 3-oxide radical (PTIO•), was introduced to further assess the antioxidant properties of FPRM. When the Fe^2+^ concentration reached 4 μg/ml, FPRM was observed to possess exceptional free radical-scavenging efficacy, achieving an efficiency of over 70% (Fig. 2O and fig. S11). In addition, with the help of a hydroxyl radical (·OH) detection kit, the experiment confirmed that FPRM also had a good scavenging ability for hydroxyl radicals (·OH) (figs. S12 and S13). Collectively, it was clear that FPRM could serve as an efficient antioxidant for scavenging ROS.

### Characterization of biological properties in vitro of FPRM

The biocompatibility of FPRM was evaluated using HUVEC cells and RAW 264.7 cells, respectively (figs. S14 and S15). The data demonstrated that both HUVEC and RAW 264.7 cells maintained high viability at concentrations up to 160 μg/ml. These results demonstrated that FPRM possessed excellent biocompatibility, laying a substantial basis for its future application-oriented investigations. MTT assay was also used to select the best LIFU irradiation condition (fig. S16). The results showed that irradiation at 3 W/cm^2^ and 1 MHz for 60 seconds using LIFU had little effect on RAW 264.7 cells (survival rate > 90%). Therefore, this condition was used in subsequent experiments.

**Fig. 3.**
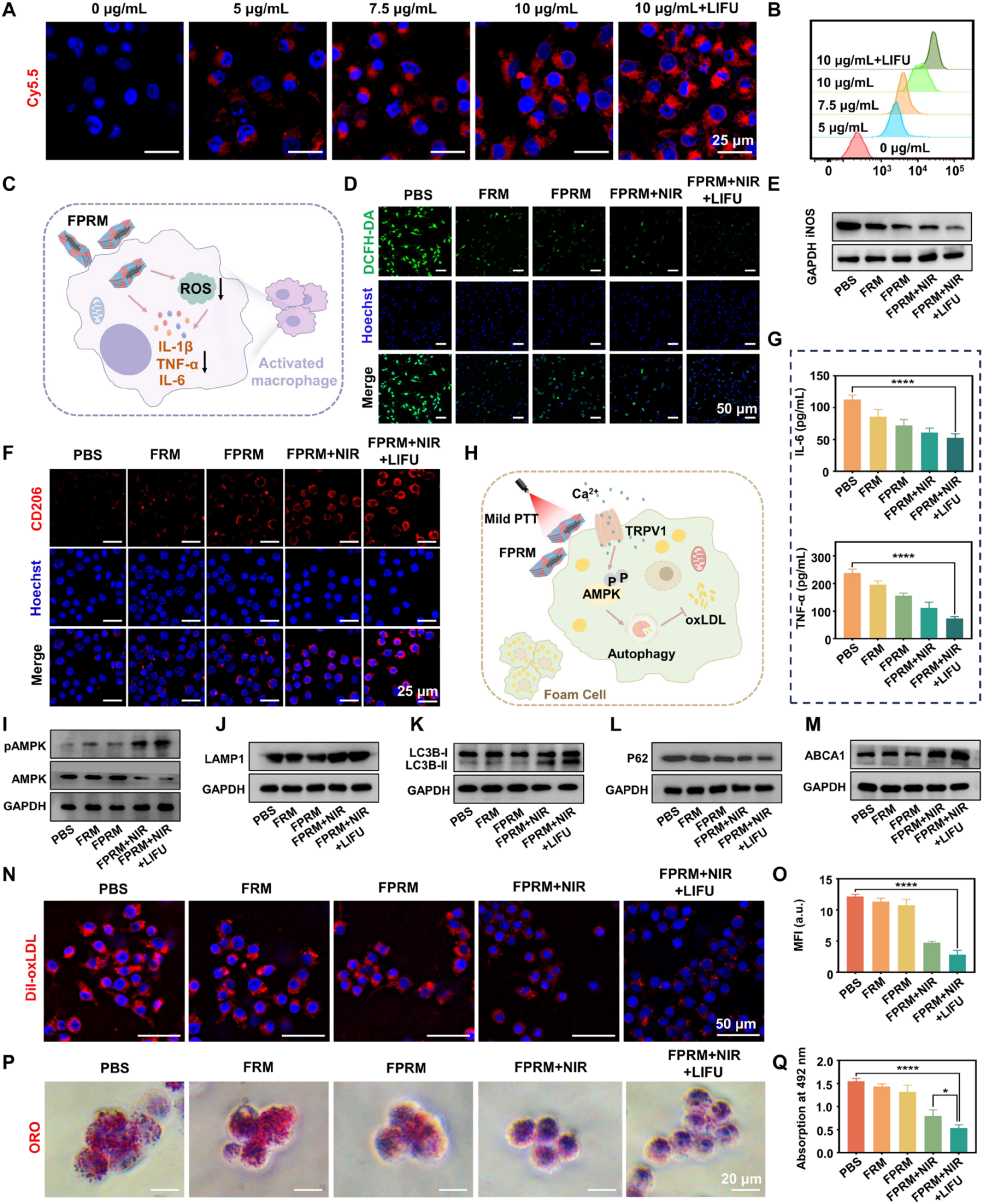
In vitro cellular investigations. (**A**) CLSM images and (**B**) FCM analysis of cellular uptake of Cy5.5-labeled FPRM by LPS-pretreated RAW 264.7 cells. Scale bar, 25 μm. (**C**) The schematic diagram illustrated the ROS eliminating and inflammation alleviating capacity of FPRM. (**D**) CLSM images of ROS levels in LPS-pretreated RAW 264.7 cells after various treatments: PBS, FRM, FPRM, FPRM + NIR, and FPRM + NIR + LIFU. Scale bar, 50 μm. (**E**) WB analysis of iNOS protein expression in LPS-stimulated macrophages after different treatments described in (D). (**F**) CLSM images of CD206 in LPS-pretreated RAW 264.7 cells following different treatments described in (D). Scale bar, 25 μm. (**G**) Levels of IL-6 and TNF-α in LPS-stimulated RAW 264.7 cells after treatments described in (D) (*n* = 3 independent experiments). (**H**) Schematic illustrating the photothermal activation of TRPV1 via the FPRM-TRPV1 switch to alleviate atherosclerosis. (**I**) WB analysis of AMPK phosphorylation in LPS-pretreated RAW 264.7 cells. WB analysis of autophagy-related proteins: (**J**) LAMP1, (**K**) LC3-II/I ratio, and (**L**) p62 in oxLDL-loaded macrophages after different treatments. (**M**) WB showing ABCA1 expression in oxLDL-pretreated RAW 264.7 cells following treatments described in (D). (**N**) CLSM images of RAW 264.7 cells pre-treated with Dil-oxLDL and then subjected to different conditions and (**O**) the corresponding MFI quantitative analysis (*n* = 3 independent experiments). Scale bar, 50 μm. (**P**) Optical microscopy images and (**Q**) the corresponding quantitative analysis of lipid in oxLDL-pretreated RAW 264.7 cells following various treatments (*n* = 3 independent experiments). Scale bar, 20 μm. Statistical comparisons were conducted using one-way ANOVA. ****P < 0.0001 and *P < 0.05. Data were presented as means ± SD.

Effective cellular uptake of drugs is a prerequisite for good therapeutic outcomes. Therefore, LPS was firstly used to induce M1 macrophages. Meanwhile, confocal laser scanning microscope (CLSM) (Fig. 3A and fig. S17) and flow cytometry (FCM) (Fig. 3B) were employed to analyze the cellular uptake of FPRM. The results demonstrated that after treating M1 macrophages with FPRM, the fluorescence signal increased in a concentration-dependent manner, with the maximum intracellular fluorescence intensity observed in the 10 μg/ml + LIFU group. This indicated that M1 macrophages possessed excellent FPRM uptake capability, and the absorption of FPRM further increased significantly when combined with LIFU.

Furthermore, congenital endothelial cell abnormalities, accompanied by persistent lipoprotein deposition and non-resolving inflammation, greatly promote the pathogenesis of AS. In the inflammatory microenvironment rich in specific pro-inflammatory mediators, the biological signaling axis formed by the interaction of vascular cell adhesion molecule-1 (VCAM-1)/integrin α4β1 promotes the recognition and endocytosis of macrophage-membrane-coated nano-drugs by endothelial cells in an inflammatory state. Based on this, we utilized HUVEC cells to evaluate the impact of modified macrophage membranes (MMs) on cellular uptake. HUVEC cells activated with lipopolysaccharide (LPS) overexpress VCAM-1 (*25*), enabling specific binding to integrin α4β1 on macrophage surfaces. We visually observed the cellular uptake of Cy5.5-labeled FPR and Cy5.5-labeled FPRM via CLSM. The results showed that FPRM exhibited a greater degree of internalization in activated endothelial cells than FPR, as evidenced by its more intense red fluorescence (figs. S18 and S19). This phenomenon indicated that FPRM had a stronger cell-internalizing ability, and further strongly confirmed that FPRM had good inflammatory chemotaxis. To further clarify the binding mechanism, after blocking VCAM-1 on activated HUVEC cells with the VCAM-1 antibody, the internalization of FPRM by activated HUVECs was significantly reduced (figs. S20 and S21). This indicated that VCAM-1 expression on HUVECs was essential for the interaction and cellular uptake of nano-drugs coated with macrophage membranes. Overall, the modification of the macrophage membrane promoted the internalization of FPRM by activated endothelial cells, demonstrating the feasibility of this strategy for targeted drug delivery in AS.

### ROS scavenging, anti-inflammatory effection and modulation of lipid metabolism of FPRM in vitro

The process of FPRM eliminating ROS and alleviating inflammation was shown in Fig. 3C. To investigate the ROS-scavenging and anti-inflammatory properties of FPRM, we used M1 macrophages (LPS-pretreated RAW264.7 cells) as an in vitro model. Compared to the control group which exhibited high ROS level, all treatment groups showed a markedly attenuated green fluorescence signal (Fig. 3D and fig. S22). The FPRM+NIR + LIFU group demonstrated the greatest ROS-scavenging capacity among all treatment groups.

To validate FPRM-induced macrophage reprogramming, we confirmed via Western blot that the expression of iNOS (a marker for M1 macrophages) was significantly decreased in cells treated with FPRM+NIR + LIFU (Fig. 3E and fig. S23). Additionally, the immunofluorescence results showed that the proportion of CD206 (a marker for M2 macrophages) increased in cells treated with FRM, which might be due to FeS inducing M2 polarization of macrophages by scavenging ROS, thereby reducing the number of M1 macrophages. The proportion of CD206 further increased in cells treated with FPRM, which might be attributed to the anti-inflammatory capabilities of the anti-inflammatory peptides BMAP-27, helping to inhibit M1 polarization of macrophages. Meanwhile, the FPRM+NIR + LIFU group showed the most significant immunofluorescence intensity of the CD206 protein (Fig. 3F and fig. S24). In addition, to investigate the anti-inflammatory capacity of FPRM, we also quantified the levels of inflammation-related cytokines. As shown in Fig. 3G and fig. S25, the FPRM+NIR + LIFU group exhibited the most pronounced reduction in the levels of interleukin 6 (IL-6), tumor necrosis factor α (TNF-α), and interleukin 1β (IL-1β) relative to all other groups. Overall, our research findings indicated that the nanoplatform demonstrated remarkable ROS-scavenging and anti-inflammatory capabilities.

The process of FPRM regulating of lipid metabolism was shown in Fig. 3H. After macrophages internalize modified lipoproteins (e.g., oxLDL), they transform into foam cells, which then adhere to the vascular endothelial cell layer. This is a key indicator of AS progression. We explored FPRM’s potential to regulate lipid metabolism in foam cells. To investigate whether the local mild photothermal effect generated by FPRM can activate the TRPV1 channel, we focused on AMP-activated protein kinase (AMPK), which played a key mediating role in the TRPV1 signaling pathway. After NIR irradiation was applied to foam cells pre-treated with FPRM, the phosphorylation level of AMPK increased significantly (Fig. 3I and fig. S26). Given the established role of AMPK phosphorylation in initiating autophagy via recruitment of downstream proteins, and the known function of macrophage protective autophagy in promoting cholesterol efflux to alleviate atherosclerosis, we delved into FPRM-induced autophagy. WB results showed that after NIR treatment, the LAMP1 (Fig. 3J and fig. S27) and LC3-II/I ratio (Fig. 3K and fig. S28) expression rose, and p62 (Fig. 3L and fig. S29) expression fell in foam cells, confirming that local temperature elevation could induce autophagy. Further WB analysis indicated that ABCA1 (Fig. 3M and fig. S30) expression was much higher in the FPRM+NIR + LIFU group than in the control group.

Next, to verify the ability to reverse cholesterol transport of the nanoplatform, we used CLSM to observe foam cells (RAW 264.7 cells pretreated with DiI-labeled oxLDL) after different treatments (Fig. 3, N and O). After FPRM+NIR or FPRM+NIR + LIFU treatment, residual oxLDL level dropped markedly, with the FPRM+NIR + LIFU group showing the best results. This proved the effectiveness of FPRM+NIR + LIFU in reversing cholesterol transport. In addition, the intracellular lipid levels, as assessed by Oil Red O (ORO) staining, were consistent with the CLSM observations (Fig. 3, P and Q). In summary, the notable regulatory impact of FPRM+NIR + LIFU on foam cell lipid metabolism highlighted the potential of the nanoplatform in reversing AS.

### Targeting ability and the PAI/MRI capacity in atherosclerotic mice of FPRM

**Fig. 4.**
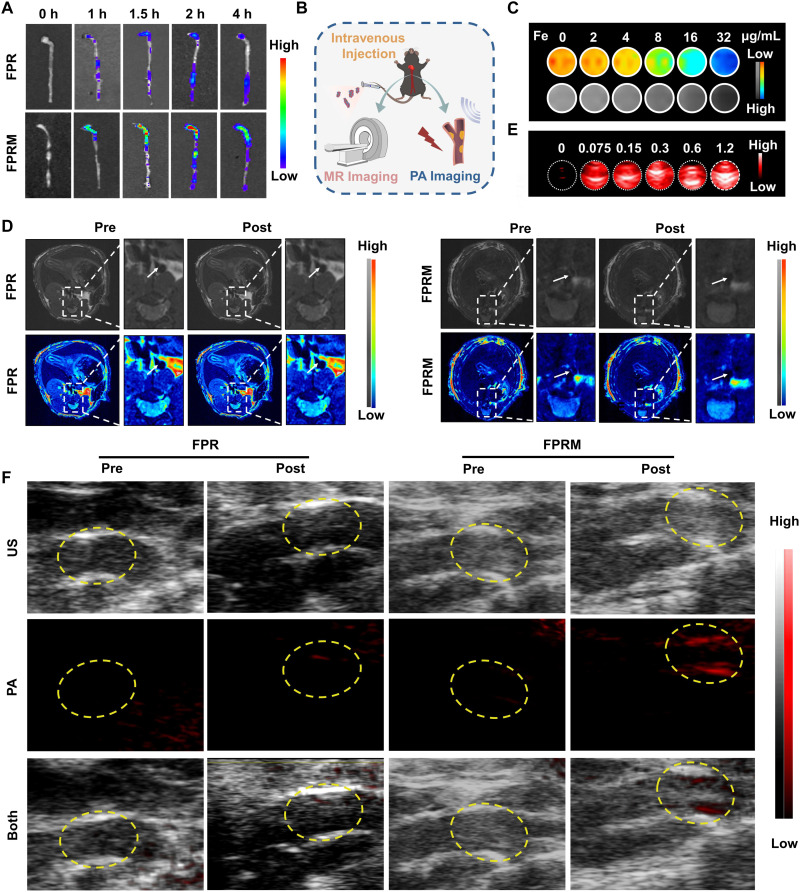
In vivo targeting and PA/MR imaging ability of FPRM in ApoE^−/−^ mice. (**A**) Ex vivo aortic imaging in atherosclerotic mice was performed at various time points (pre, 1, 1.5, 2, and 4 hours) following the intravenous administration of Cy5.5-labeled FPR or FPRM. Aortas were dissected and imaged to evaluate the time-dependent accumulation of nanosheets in atherosclerotic plaques. (**B**) Scheme of FPRM for PA imaging and MR imaging of atherosclerotic plaques. (**C**) T2-weighted images and pseudo-color of the MR signal intensity of FPRM at varying Fe concentrations (0, 2, 4, 8, 16, 32 μg/ml) in RAW 264.7 cells pre-stimulated with LPS. (**D**) At designated time points (pre- and 2 hours post-injection), T2-weighted MR and pseudo-color images of atherosclerotic mice were acquired following the separate intravenous administration of FPR or FPRM. (**E**) In vitro PA images of FPRM solutions at varying Fe concentrations (0, 0.075, 0.15, 0.3, 0.6, 1.2 mg/ml). (**F**) In vivo PA images of the plaque region acquired from atherosclerotic mice before and 2 hours after intravenous injection of FPR or FPRM, respectively. h, hours.

Fluorescently labeled nanomedicine can be used to assess the accumulation and specificity of drugs in target tissues, along with the delivery and distribution of drugs in vivo. In this study, FPR and FPRM were assigned as the control and experimental groups, respectively, to evaluate their in vivo targeting capabilities in Atherosclerotic Mice (Fig. 4A and figs. S31 to S33). The results showed that the fluorescence labeling of FPRM revealed significant accumulation in atherosclerotic regions, which was much higher than the accumulation of the FPR group in these regions. This finding indicated that the hybrid membrane, due to the inflammatory chemotactic ability of the macrophage membrane, possessed excellent atherosclerosis targeting ability, providing robust support for its precise in vivo delivery and therapeutic effectiveness. Additionally, the fluorescence intensity of FPRM peaked at 2 hours post-administration and subsequently declined, reflecting the progressive metabolism and clearance of the labeled nanomedicine. To further investigate whether LIFU could enhance the local accumulation of FPRM in plaque regions, additional atherosclerotic mice were intravenously injected with Cy5.5-labeled FPRM and treated with or without LIFU at 1 hour post-injection. Ex vivo fluorescence imaging of isolated aortas collected at 2 hours post-injection showed that the FPRM + LIFU group exhibited stronger fluorescence signals in the atherosclerotic aorta than the FPRM group (figs. S34 and S35), suggesting that LIFU could further promote the local accumulation of FPRM in plaque lesions. Based on the impressive atherosclerotic plaque-targeting enrichment properties of FPRM, the PA and MR imaging capabilities of FPRM were further investigated (Fig. 4B). Magnetic resonance imaging (MRI), with its excellent deep tissue penetration ability and good spatial resolution, has become a widely popular imaging modality in clinical applications. As shown in Fig. 4C, the results of M1 macrophages (LPS-pretreated RAW cells) revealed that a gradual darkening of the MR images was observed with increasing concentration. The vitro experiments demonstrated the capability of FPRM to enhance T2-weighted MRI. Subsequently, we further evaluated the MRI properties of FPRM in vivo (Fig. 4D and fig. S36). As expected, in the FPRM group, after tail vein injection of FPRM, the MRI signal in the plaque area significantly darkened, indicating that FPRM had a good effect in T_2_-weighted MRI and was superior to the FPR group. Photoacoustic imaging (PAI) is a promising imaging modality due to its non-invasiveness and high sensitivity. The significant NIR absorption capacity of FPRM suggested its utility as a highly effective contrast agent for photoacoustic imaging. Prior to in vivo PA imaging, the PA properties of FPRM were first characterized in vitro (Fig. 4E). A strong linear correlation (R^2^ = 0.9987) was observed between the PA intensity and FPRM concentration, demonstrating a clear dose-dependent response (fig. S37). The PA imaging capability of FPRM and FPR was further verified in a mouse model of atherosclerosis (Fig. 4F and fig. S38). PA images were acquired 2 hours after drug administration, with subsequent quantitative analysis of the signal intensity. Prior to administration, plaque areas in both groups exhibited no obvious PA signals. However, in contrast to the FPR group, the plaque region in the FPRM group exhibited a markedly more intense PA signal after injection. This enhancement stemmed from the targeting effect of FPRM on the plaque area conferred by the inflammatory chemotactic ability of the macrophage membrane. These findings provided strong evidence for the efficient accumulation of FPRM in the plaque area and its potential as a PAI contrast agent. The all above results indicated that FPRM had a high accumulation efficiency in the plaque areas and could be used for bimodal imaging to achieve visual monitoring.

### Atherosclerotic therapy with FPRM in vivo

**Fig. 5.**
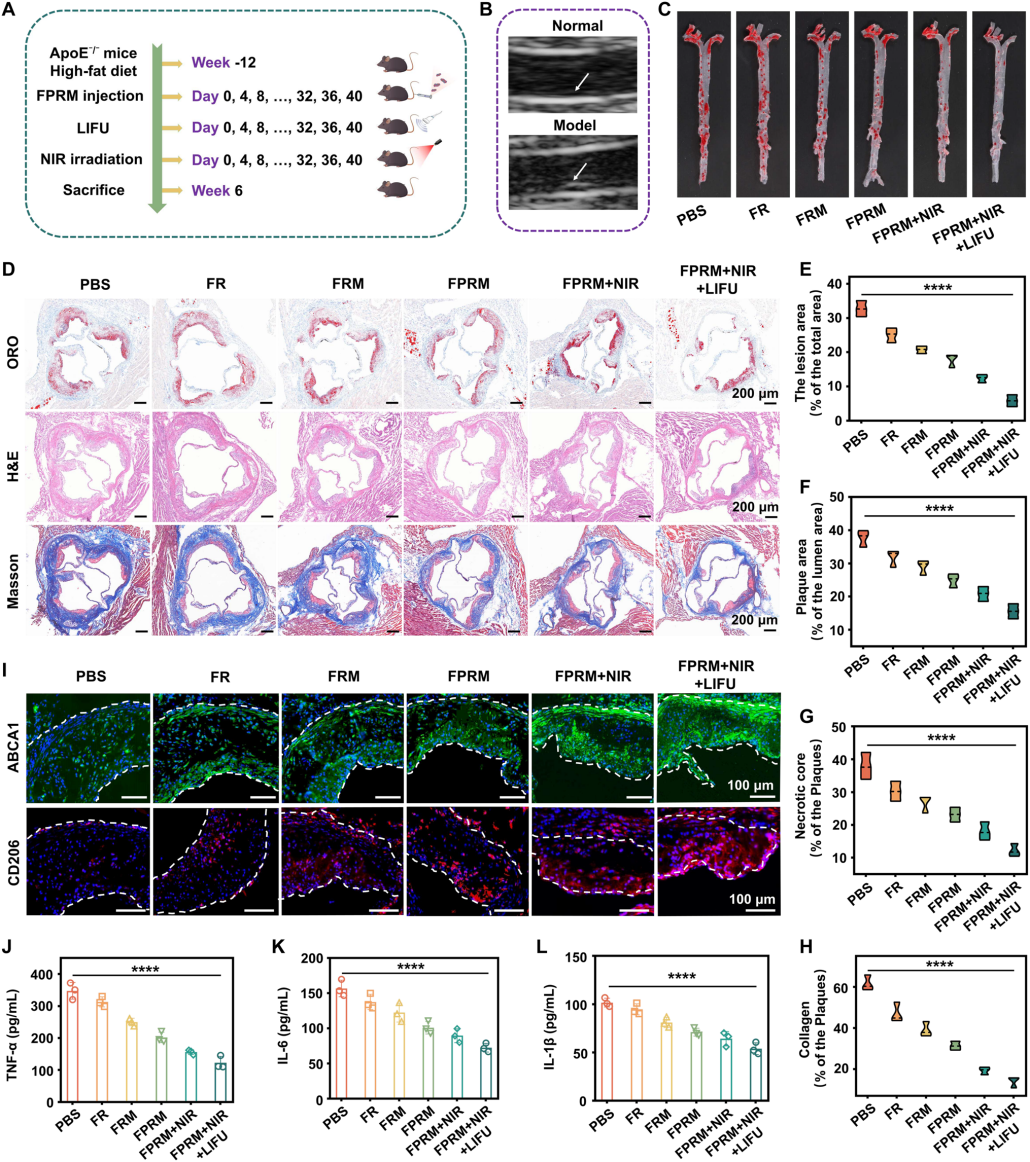
In vivo therapeutic effects of FPRM in ApoE^−/−^ mice. (**A**) Schematic illustration of the atherosclerosis model establishment and subsequent therapeutic regimen in ApoE^−/−^ mice. (**B**) Ultrasound images of atherosclerotic plaques in normal and successfully modeled ApoE^−/−^ mice. (**C**) Representative en face aortic images with ORO staining from mice receiving different treatments: PBS, FR, FRM, FPRM, FPRM+NIR, and FPRM+NIR + LIFU. (**D**) Representative aortic sections stained with ORO, H&E, and Masson’s trichrome following various treatments described in (C). Scale bars, 200 μm. (**E**) Quantification of the ORO-positive lesion area relative to the total aortic surface area (*n* = 3 biologically independent mice). Quantification of: (**F**) plaque area, (**G**) necrotic core, and (**H**) collagen deposition in aortic sections across treatment groups (*n* = 3 biologically independent mice). (**I**) Representative aortic sections immunostained for ABCA1 and CD206 following different treatments described in (C). Scale bars, 100 μm. Levels of Inflammation related cytokines (**J**) TNF-α, (**K**) IL-6, and (**L**) IL-1β in serum, measured by ELISA (*n* = 3 biologically independent mice). Statistical comparisons were conducted using one-way ANOVA. ****P < 0.0001. Data were presented as means ± SD.

Based on the promising results of FPRM in vitro, we further evaluated its in vivo therapeutic efficacy in a comorbid mouse model of atherosclerosis and hepatic steatosis. The model and the corresponding treatment regimen were illustrated in Fig. 5A. We first validated the establishment of the model using aortic and hepatic ultrasound imaging. Fig. 5B showed ultrasound images of the abdominal aortic wall in normal mice and ApoE^−/−^ mice after model establishment. In the model group, significant thickening of the aortic intima-media accompanied by plaque formation was observed, confirming successful modeling of atherosclerosis. Simultaneously, hepatic ultrasound images revealed increased echogenicity along with elevated liver stiffness (fig. S39) in the model group, indicating concurrent development of hepatic steatosis. Accordingly, all subsequent vascular and hepatic evaluations in this study were performed using the same cohort of comorbid model mice. To verify whether the applied NIR irradiation could generate an effective local mild photothermal effect in ApoE^−/−^ mice in vivo, we further performed infrared thermal imaging during laser treatment. Under 808-nm irradiation (1.0 W/cm^2^, 5 min), the FPRM-treated mice showed a clear local temperature increase in the abdominal aortic plaque region, with the temperature reaching approximately 45°C, which fell within the mild photothermal range and was sufficient to support activation of the TRPV1-related pathway (fig. S40). Subsequently, Atherosclerotic ApoE^−/−^ mice were subjected to treatment with PBS (control), FR, FRM, FPRM, FPRM+NIR, or FPRM+NIR + LIFU, respectively. At the experimental endpoint, the aortas were harvested and longitudinally incised, followed by ORO staining to visualize atherosclerotic plaques (Fig. 5C and fig. S41), and the quantitative results of plaque area were shown in Fig. 5E. The FPRM + NIR + LIFU treatment, which possessed effective targeting, antioxidant, anti-inflammatory, and mild photothermal lipid-regulating capabilities, showed the best efficacy. It led to a significant reduction in aortic plaque distribution. Immunohistochemical analysis of aortic sections via ORO, hematoxylin-eosin (H&E), and Masson staining showed that FPRM+NIR + LIFU treatment significantly inhibited the progression of AS (Fig. 5D), which was specifically manifested as the lowest percentage of plaque area (Fig. 5F), reduced formation of necrotic cores (Fig. 5G), and decreased collagen deposition (Fig. 5H).

To gain deeper insights into the impact of FPRM+NIR + LIFU on the progression of AS at the molecular level, we conducted fluorescence staining to assess the expression of ABCA1 and CD206 in aortic plaques. ABCA1, a key molecule involved in lipid efflux, and CD206, a specific M2 macrophage marker, were both selected as detection indicators. As presented in Fig. 5I, in contrast to the control group, the fluorescence intensity of ABCA1 (green) (fig. S42) and CD206 (red) (fig. S43) in the aortic plaques of mice in the FPRM+NIR + LIFU group was significantly enhanced. The results indicated that in atherosclerotic mice, FPRM+NIR + LIFU could enhance lipid efflux and drive the polarization of macrophages towards the M2 type, which was consistent with the findings at the cellular level. Meanwhile, enzyme-linked immunosorbent assay (ELISA) kit analysis of serum inflammatory factors (TNF-α, IL-6, IL-1β) in Fig. 5, J to L, demonstrated that FPRM + NIR + LIFU treatment had remarkable anti-inflammatory effects, with reduced secretion of pro-inflammatory factors. In summary, FPRM+NIR + LIFU treatment could effectively promote lipid efflux, reduce inflammatory responses, and inhibit the progression of atherosclerosis.

### Attenuation of hepatic steatosis with FPRM and investigation of FPRM’S in vivo biosafety

**Fig. 6.**
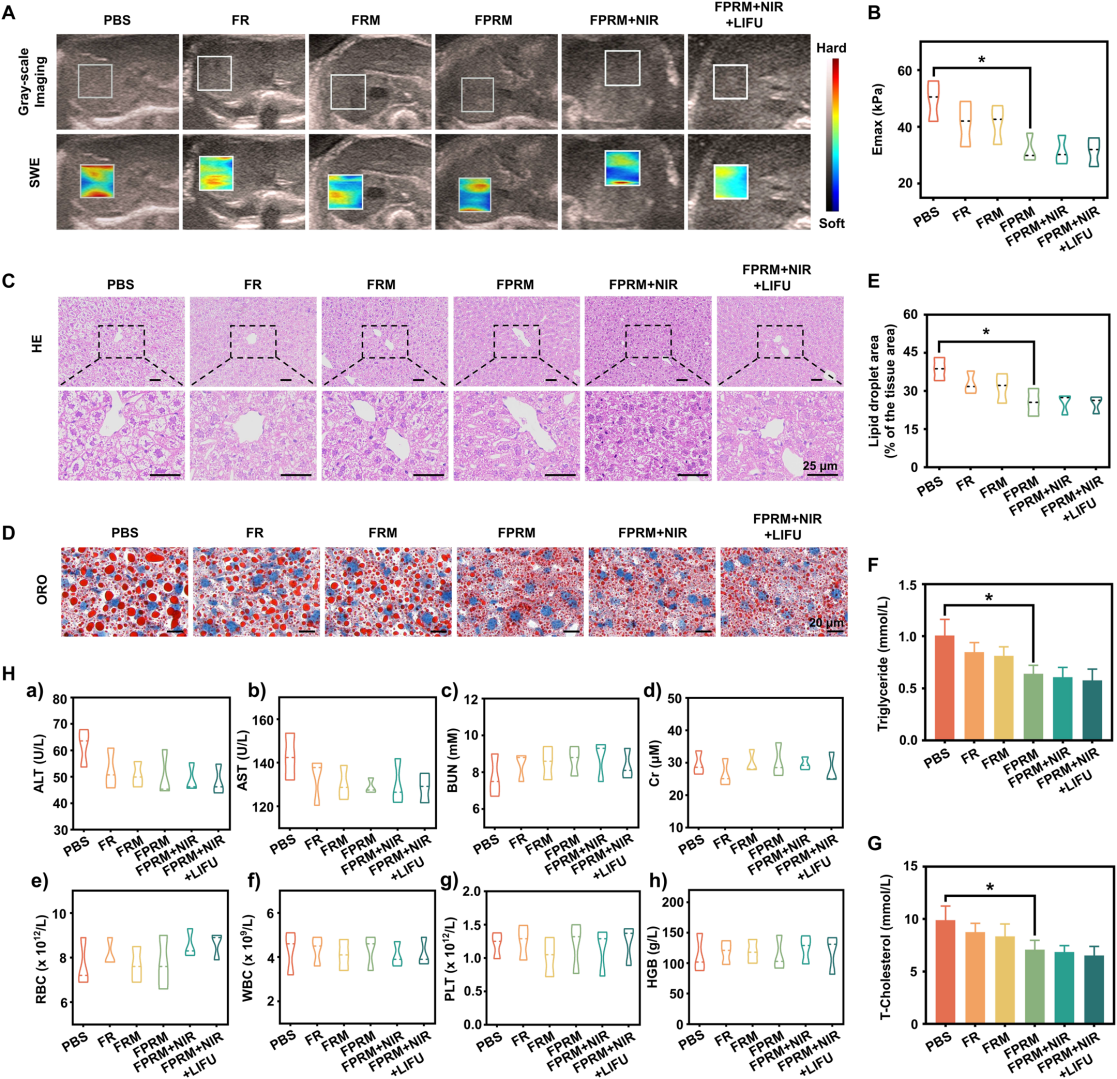
In vivo attenuation of hepatic steatosis and biosafety profile of FPRM. (**A**) Gray-scale images and SWE images of livers in different treatment groups (PBS, FR, FRM, FPRM, FPRM+NIR, and FPRM+NIR + LIFU). (**B**) The mean Young’s modulus (Emax) of liver tissues in each group was determined by ultrasound SWE. (**C**) H&E staining of liver sections from each group. Scale bar, 25 μm. (**D**) Oil Red O staining of liver sections from each group (Scale bar, 100 μm) and (**E**) the corresponding quantification (*n* = 3 biologically independent mice). Detection results of serum (**F**) Triglyceride and (**G**) T-Cholesterol levels (*n* = 3 biologically independent mice). (**H**) Blood biochemistry and routine blood analysis of atherosclerotic mice after different treatments (*n* = 3 biologically independent mice). Statistical comparisons were conducted using one-way ANOVA. *P < 0.05. Data were presented as means ± SD.

We further evaluated the nanoplatform’s therapeutic efficacy against hepatic steatosis. As shown in Fig. 6, A and B, the FPRM + NIR + LIFU group exhibited a reduction in echogenic fat signals on grayscale liver ultrasound, and also showed a decrease in liver stiffness on shear-wave elastography (SWE), indicating some alleviation of hepatic steatosis. Histologically, H&E staining in Fig. 6C showed restored, orderly hepatocyte architecture with diminished vacuolar degeneration, and Oil-Red-O staining in Fig. 6, D and E, revealed a reduction in lipid droplets, yielding the lowest quantified lipid-area fraction. Consistent with imaging and morphological outcomes, serum triglyceride (TG) and total cholesterol (TC) levels were decreased to some extent (Fig. 6, F and G). In summary, the FPRM + NIR + LIFU strategy efficiently alleviated hepatic steatosis by suppressing oxidative stress and inflammation.

An ideal therapeutic regimen should achieve robust efficacy without overt systemic toxicity. As illustrated in fig. S44, hemolysis remained <5% even at FPRM concentrations up to 200 μg ml^−1^, underscoring its excellent blood compatibility. Subsequent serum biochemistry and complete blood count analyses (Fig. 6H) revealed no significant differences in any parameter across groups and within physiologically acceptable limits, suggesting the absence of any detrimental impact on overall physiological function. Post-treatment histological analysis of major organs (Fig. 6C and fig. S45) showed no discernible histopathological damage, affirming the excellent biosafety profile of the nanoplatform in vivo.

## DISCUSSION

Atherosclerosis is a primary driver of global cardiovascular morbidity and mortality. However, current therapeutic strategies are largely confined to managing isolated pathological processes, such as hyperlipidemia or local vascular inflammation. These approaches often overlook the systemic metabolic dysregulation frequently associated with atherosclerosis, particularly hepatic steatosis, which has been increasingly recognized as a critical comorbidity and driver of disease progression.

To our knowledge, we reported for the first time an intelligent nanoplatform capable of synergistically regulating AS and hepatic steatosis. This nanoplatform was constructed by BMAP-27 with significant anti-inflammatory activity onto FeS nanosheets, followed by coating with the RMM to achieve specific targeting of atherosclerotic plaques. Under the cavitation effect induced by LIFU, FPRM precisely accumulated in the plaque region, transiently enhanced cell membrane permeability, and significantly improved drug uptake and retention at the lesion site.

Upon successful targeting and penetration into the plaque, NIR irradiation enabled FPRM to convert light energy into heat, generating a localized mild photothermal effect. This activated the thermosensitive TRPV1 channels on foam cells, allowing Ca^2+^ influx. The rise in cytosolic Ca^2+^ activated autophagy in foam cells, upregulated ABCA1-mediated cholesterol efflux, and reduced oxLDL accumulation. Concurrently, the FeS nanosheets efficiently scavenged various ROS excessively accumulated within the plaques, effectively alleviating oxidative stress. This antioxidant effect worked synergistically with the anti-inflammatory activity of the loaded BMAP-27 to suppress AS-related inflammation, promote the polarization of macrophages towards the reparative M2 phenotype, and achieve effective remodeling of the plaque inflammatory microenvironment.

Unexpectedly, the most classic liver accumulation and metabolism pathway of the nanoplatform had also become an advantage. In the liver, its potent ROS-scavenging capacity and anti-inflammatory effects were similarly exerted, effectively alleviating inflammatory injury and hepatocyte steatosis, thereby achieving systemic metabolic reprogramming. This simultaneous intervention against both AS and its critical comorbidity, hepatic steatosis, represented a “cardio-hepatic co-therapy” approach that embodied a systemic therapeutic strategy, offering a previously unknown pathway for enhancing the overall efficacy of AS treatment.

Furthermore, the inherent PA and MR imaging capabilities of FPRM constituted a complete theranostic platform, enabling non-invasive, real-time monitoring of in vivo drug distribution, plaque targeting, and therapeutic response, thereby laying the foundation for future personalized precision medicine.

Certainly, the clinical translation of this research still faces some challenges. For instance, the large-scale, standardized preparation process of the hybrid membrane requires further optimization, and the long-term metabolic fate and biocompatibility of the nanoplatform need more comprehensive evaluation in large animal models.

Collectively, we constructed an intelligent theranostic platform founded on biomimetic FeS nanosheets. This nanoplatform ensured effective enrichment of the drug in AS plaques through active targeting and LIFU-enhanced penetration, promoted cholesterol efflux via photothermal activation of the TRPV1-autophagy axis, and remodeled the plaque microenvironment through synergistic antioxidant/anti-inflammatory effects. Simultaneously, it alleviated hepatic steatosis by leveraging its natural liver metabolic pathway, thereby achieving synergistic treatment of atherosclerosis and its systemic metabolic risk factors. This work not only provided a previously unexplored solution for AS treatment, but also offered insights for developing systemic intervention strategies against other complex chronic diseases.

## MATERIALS AND METHODS

### Intracellular ROS scavenging detection

After being plated in confocal dishes, RAW264.7 cells were treated with LPS (1 μg/ml) for 24 hours. Subsequently, the cells were subjected to the following treatments: PBS, FRM, FPRM, FPRM + NIR, and FPRM + NIR + LIFU. The NIR parameters were set at 808 nm, 1.0 W/cm^2^ for 5 min and the LIFU parameters were set at 1.0 MHz, 3 W/cm^2^ for 60 s. After the treatment, the culture medium was removed and replaced with the ROS detection kit solution containing DCFH-DA (2,7-dichlorodihydrofluorescein, final concentration 20 μM). The cells were then incubated for 30 minutes. Finally, the cells were observed using the CLSM, and the mean fluorescence intensity (MFI) was measured.

### Detection of macrophage polarization in vitro

We seeded RAW264.7 cells in confocal dishes and stimulated them with LPS (1 μg/ml) for 24 hours. Then, the cells were treated as follows: (PBS, FRM, FPRM, FPRM + NIR, FPRM + NIR + LIFU). Following three PBS washes, the cells were fixed with 4% paraformaldehyde for 30 minutes. Subsequently, we treated these cells with a membrane-breaking solution containing 1% Triton. After 30 minutes of treatment, the cells were rinsed three times with PBS and then treated with diluted anti-CD206 antibody overnight in the dark. Following three more washes with PBS, the cells were exposed to diluted fluorescent secondary antibodies for 1 hour under dark conditions. Following another three PBS washes, the cell nuclei were counterstained with Hoechst. Finally, the cells were imaged by CLSM and the corresponding MFI was quantified.

### Cholesterol efflux assay in vitro

RAW264.7 cells were seeded into 6-well plates and treated with ox-LDL (50 μg/ml) for 24 hours. The cells were then subjected to the following treatments: PBS, FRM, FPRM, FPRM + NIR, and FPRM + NIR + LIFU. Post-treatment ORO staining was performed, followed by visualization with an inverted fluorescence microscope. Meanwhile, RAW264.7 cells were plated in confocal dishes and stimulated with DiI-labeled ox-LDL for 24 hours. Then, the cells were treated as follows: PBS, FRM, FPRM, FPRM + NIR, and FPRM + NIR + LIFU. Subsequently, the cell nuclei were labeled with 100 μl of DAPI. Finally, the cells were observed by CLSM, and the MFI was calculated.

### Ethics approval

All animal experiments were approved by the Ethics Committee of Shandong First Medical University Affiliated Cancer Hospital, with the number of SDTHEC2023003137.

### In vitro and in vivo PA imaging

PA imaging was all conducted using the Vevo LAZR-X system (VisualSonics Inc., New York, NY). To evaluate the PA performance of FPRM in vitro, FPRM solutions with different concentrations (0, 0.075, 0.15, 0.3, 0.6, 1.2 mg/ml) were injected into PA imaging tubes, and the PA imaging efficiency of FPRM at different concentrations was analyzed. To evaluate the imaging of FPRM on atherosclerosis in vivo, the atherosclerotic mice were randomly assigned to two groups and intravenously injected with FPR or FPRM (8 mg/kg). PA images were acquired and the PA signals of the plaque regions were recorded before injection and 2 hours post-injection.

### Therapeutic effect on atherosclerotic mice

The atherosclerotic mice were randomly assigned to different groups (*n* = 3 mice per group) for subsequent treatments, including: PBS, FR, FRM, FPRM, FPRM+NIR, FPRM+NIR + LIFU. A dosage of 8 mg/kg (Fe) was delivered via tail vein injection every 4 days. The NIR parameters were set at 808 nm, 1.0 W/cm^2^ for 5 min and the LIFU parameters were set at 1.0 MHz, 3 W/cm^2^ for 60 s. After the different treatments, the atherosclerotic mice were euthanized. Following excision, the entire aortic tract (from the arch to the abdominal aorta) was stained with ORO for plaque area evaluation. The aortic plaque area of each sample was calculated using ImageJ software. The aortic root was immunostained with anti-ABCA1 and anti-CD206 antibodies for semi-quantitative analysis of the respective receptor expression levels. Aortic root sections were subjected to a series of histological stains (Oil Red O, H&E, and Masson’s trichrome) for the evaluation of necrotic core formation and collagen deposition within atherosclerotic plaques. Serum levels of TNF-α, IL-6, and IL-1β were quantified by ELISA kits. To comprehensively assess the impact of various therapeutic interventions on the livers of mice, we employed a variety of experimental techniques. First, we used ultrasound imaging technology to perform two-dimensional imaging and shear - wave elastography of the mouse liver to monitor changes in liver morphology and stiffness. In addition, we conducted histological analyses of the mouse livers, including H&E staining and ORO staining, to evaluate structural changes and lipid deposition in the liver. Finally, we collected blood samples from the mice and measured the levels of TG and TC to assess lipid metabolism status.

### Statistical analysis

Data analysis was performed using GraphPad Prism and Origin software. All data were presented as mean ± standard deviation (Mean ± SD). Statistical comparisons were conducted using either one-way ANOVA or two-way ANOVA. A *P* value of < 0.05 was considered to indicate statistical significance, with specific notations as follows: ns (*P* > 0.05), * (*P* < 0.05), ** (*P* < 0.01), *** (*P* < 0.001), and **** (*P* < 0.0001).

## Supplementary Materials

### This PDF file includes:

Supplementary Text Figs. S1 to S45 References
