# Supplementary material for: Engineered heart-liver axis nanoregulators synergize autophagy activation and systemic metabolic reprogramming against atherosclerosis
Source: Sci Adv. 2026 Jul 31;12(31):eaee3352. doi: 10.1126/sciadv.aee3352 (PMC13426405; doi:10.1126/sciadv.aee3352)
Supplement: Supplementary file 1 — Supplementary Text Figs. S1 to S45 References [file sciadv.aee3352_sm.pdf]

Science Advances
AAAS

Supplementary Materials for

Engineered heart-liver axis nanoregulators synergize autophagy activation and systemic metabolic reprogramming against atherosclerosis

Yiyong Tang *et al.*

Corresponding author: Xiao Sun, sunxiao@sdfmu.edu.cn; Houren Zhou, hour1666666@163.com; Weihai Xu, xuwh@pumch.cn; Jun Wu, wujun108@sina.com

Sci. Adv. **12**, eaee3352 (2026)
DOI: 10.1126/sciadv.aee3352

This PDF file includes:

Supplementary Text
Figs. S1 to S45
References

Materials

Ammonium iron (II) sulfate hexahydrate, thioacetamide, polyethylene glycol (PEG), trisodium citrate, polyethyleneimine (PEI), and H33342 trihydrochloride (Hoechst 33342) were supplied by Aladdin Biochemical Technology Co., Ltd. (Shanghai, China). Phosphate-buffered saline (PBS) and penicillin-streptomycin solution were purchased from Beyotime Biotechnology Co., Ltd. (Shanghai, China). The BCA protein assay kit, cell lysis buffer for Western blotting, primary antibody dilution buffer, and other commonly used buffers were purchased from Aladdin Chemical Co., Ltd. (Shanghai, China). Cy5.5-NHS, DPPH, and PTIO were purchased from Shanghai Maokang Biotechnology Co., Ltd. (Shanghai, China). Lipopolysaccharide (LPS) and the BCA Protein Assay Kit were obtained from Solarbio Science & Technology Co., Ltd. (Beijing, China). The Oil Red O staining kit and 4′,6-Diamidino-2-phenylindole (DAPI) were also purchased from Solarbio Science & Technology Co., Ltd. (Beijing, China). Oxidized low-density lipoprotein (oxLDL) was obtained from Yuanye Biology (Shanghai, China). DiI-labeled oxLDL was purchased from Shanghai Yanhui Biotechnology Co., Ltd. (Shanghai, China). DCFH-DA was purchased from Beyotime Biotechnology Co., Ltd (Shanghai, China). TNF-α ELISA Kit, IL-6 ELISA Kit and IL-1β ELISA Kit were purchased from Shanghai Boersen Biotechnology Co., Ltd. (Shanghai, China). Anti-GAPDH, anti-iNOS, anti-pAMPK, anti-AMPK, anti-LC3B, anti-P62, and anti-ABCA1 were acquired from Santa Cruz Biotechnology (USA). Anti-CD206 and anti-LAMP1 were purchased from Abcam (UK). All the chemicals used in this study were analytical grade chemicals.

Synthesis of FeS

1.2 mmol of ammonium iron (II) sulfate hexahydrate and 0.4 mmol of sodium citrate dihydrate were dissolved in 15 mL of ethylene glycol. Subsequently, 0.5 g of polyethyleneimine (PEI) was added and completely dissolved. The mixture was stirred at room temperature with magnetic stirring at 800 rpm for 120 min. Thioacetamide was then added to the solution to a final concentration of 0.05 M, followed by the addition of 2 mL of triethanolamine, and the mixture was stirred at 800 rpm until dissolved. The resulting precursor solution was transferred to a Teflon-lined autoclave and heated at 200 °C for 24 hours. After the reaction, the product was centrifuged at 14000 rpm for 10 min, washed twice with anhydrous ethanol, and finally dispersed in anhydrous ethanol to obtain the final product FeS.

Preparation of Red Blood Cell Membranes (RM)

Whole blood was obtained from the orbital vein of C57BL/6 mice. First, the eyeball was enucleated, and sodium citrate solution was used as an anticoagulant. The blood was centrifuged at 4 °C and 720 g for 10 minutes. The upper plasma layer and the middle white blood cell layer were removed, and the lower red blood cell layer was collected. Subsequently, the red blood cells were washed with ice-cold PBS containing 1 mM PMSF. Pure red blood cell

membranes were obtained by removing hemoglobin through multiple hypotonic treatments, which were then aliquoted and stored at -80 °C. The membrane protein concentration was determined by the BCA method. Given that the mass ratio of phospholipids to proteins in the cell membrane is approximately 2:1 (*26*), the concentration of the phospholipid membrane was calculated accordingly.

Preparation of Macrophage Membranes (MM)

MM was isolated from RAW 264.7 cells. The membrane was obtained using the Membrane Protein Extraction kit. RAW 264.7 cells were seeded in DMEM medium supplemented with 10% (v/v) fetal bovine serum (FBS) and 1% penicillin-streptomycin, and cultured in a humidified environment with 5% CO_2 at 37 °C. When the cell confluence reached 90%, the medium was discarded and 500 μL of membrane-protein extraction reagent A containing 1 mM PMSF was added, followed by lysis on ice for 3-5 min. The lysate was scraped off with a cell scraper, transferred to a centrifuge tube, and subjected to five freeze–thaw cycles between liquid nitrogen and a 37 °C water bath to ensure complete lysis. The mixture was then centrifuged at 800 g for 10 min at 4 °C to remove nuclei, and the supernatant was collected. This supernatant was further centrifuged at 20,000 g for 15 min at 4 °C, yielding the cell membrane pellet. The cell membranes were washed with PBS, aliquoted, and stored at -80 °C. The protein concentration was determined by the BCA assay.

Preparation of FPRM

First, 2 mg of HS-PEG-NHS was dissolved in 2 mL of pure water, and 50 μg of the BMAP-27 was added. The reaction mixture was maintained under stirring in an ice bath for 20 minutes. Next, 1 mg of FeS was introduced into the system, and stirring was continued in an ice bath for 120 min to obtain FP. Afterwards, RM-MM (RMM, RM:MM mass ratio = 1:1) was added, and the dispersion was sonicated for 10 min using a SCIENTZ-IID ultrasonic cell disruptor (Scientz, China) to yield FPRM. When only the corresponding amount of RM was added, FeS/BMAP-27/RM (FPR) was obtained. Finally, FPRM and FPR were collected as pellets by centrifugation and stored at 4 °C.

Characterization

The microstructure and elemental distribution of FeS nanosheets were observed by TEM (HITACHI HT7800, Japan), and the AFM (Bruker Dimension Icon, Germany) was used to measure the thickness of the FeS nanosheets. The crystal structure measurements were performed on XRD (MiniFlex 600, Rigaku, Japan). The valence states of each element in the samples were determined by the XPS (ESCALAB 250 Xi, Thermo Scientific, USA). Fourier-transform infrared spectroscopy (FTIR, Nicolet iS5, Thermo, USA) was employed to analyze FeS, FP, and FPRM. The particle size and zeta potential were measured using a DLS detector (Zetasizer Nano S90, Malvern, UK). The transfer and retention of membrane proteins during the preparation process were evaluated by the sodium dodecyl sulfate-polyacrylamide gel

electrophoresis (SDS-PAGE). The FCRM group was compared with the RM, MM, and RMM groups, with the protein loading amount kept consistent across all groups. Metal content was quantified on an ICP-OES (G8018A, Agilent, USA).

Coomassie brilliant blue

The grafting of BMAP-27 peptides onto FeS was evaluated by SDS-PAGE. The experiment was divided into three groups: the FeS group, the BMAP-27 peptides group, and the FP group. Prior to loading, all samples were first treated with acid to fully degrade FeS, ensuring the complete release of the BMAP-27 peptides. Subsequently, the samples were added to the SDS-PAGE gel for electrophoresis. After electrophoresis, the gel was stained with Coomassie Brilliant Blue for 2 h and then repeatedly rinsed with deionized water until the background was colorless and the bands were clear. Finally, images were captured using a gel imaging system (Biorad ChemiDoc MP, Bio-Rad, USA).

SDS-PAGE evaluation of membrane protein retention and stability after NIR irradiation

SDS-PAGE was used to evaluate the retention of membrane proteins during the preparation of FPRM and to further analyze their stability after near-infrared irradiation. RM and MM were used as single-component membrane controls, while hybrid membranes (RMM) and membrane-coated nanosheets (FPRM) were used as experimental groups to assess the retention of membrane proteins during the hybridization and coating processes. To further evaluate the effect of mild photothermal conditions on the membrane protein components, the FPRM dispersion (Fe: 10 μg/mL) was irradiated with an 808 nm laser at 1.0 W/cm² for 5 min to obtain the FPRM + NIR group, which was then compared with the FPRM group without irradiation. Subsequently, membrane proteins were extracted from each group by lysis and heated at 95 °C for 5 min. Equal amounts of protein were loaded onto 10% SDS-PAGE gels for electrophoretic separation. After electrophoresis, the gels were stained with Coomassie Brilliant Blue for 2 h and then repeatedly rinsed with deionized water until the background was clear and the bands were well visualized. Finally, images were captured using a gel imaging system (Biorad ChemiDoc MP, Bio-Rad, USA). The retention of membrane proteins during the preparation of FPRM and their stability after near-infrared irradiation were evaluated by comparing the protein band patterns across the groups.

Evaluation of photothermal properties of FPRM in vitro

The ultraviolet absorption spectra of FeS nanosheets at different concentrations (calculated as Fe: 0, 10, 20, 40 μg/mL) were determined. Different concentrations of FPRM (0, 10, 20, 40 μg/mL) were exposed to an 808 nm laser (1.0 W/cm², 5 min), and the temperature was recorded every 10 seconds to investigate the photothermal capacity of FPRM nanosheets. To further validate the photothermal effects of FPRM, the temperature rise curves of FPRM aqueous dispersion were recorded under gradient power density (0.5, 1.0, 1.5 W/cm^2) at 10 μg/mL FPRM concentration. To evaluate the photothermal cycling stability of FPRM, an aqueous dispersion

with a concentration of 10 μg/mL was taken and irradiated repeatedly for 5 cycles using a near-infrared laser at 1.0 W/cm². For each cycle, the dispersion was first irradiated for 5 minutes and then allowed to cool naturally to room temperature, during which the temperature was monitored in real time.

Biocompatibility

HUVEC cells and RAW264.7 cells in logarithmic growth phase were taken and respectively inoculated in 96-well plates, and cultured in DMEM medium at 37°C with 5% CO_2. Subsequently, the cells were treated by FPRM with at different concentrations (0, 20, 40, 80, and 160 μg/mL), cultured for 48 h, and the cell viability was determined by MTT assay.

The ultrasound therapy system was operated at a fixed frequency of 1 MHz. The system was operated with exposure durations of 60 and 90 seconds, and at intensity levels of 0, 1, 2, 3, and 4 W/cm². A transducer probe was positioned directly beneath the cell culture plate to ensure efficient ultrasonic coupling. Subsequently, the culture plate was placed on the transducer surface to assess potential cellular effects of the applied ultrasound intensity.

FPRM labeled with Cy5.5

First, FeS nanosheets are dispersed in PBS to prepare a solution with a concentration of 1 mg/mL. Next, the Cy5.5-NHS solution was added at a mass ratio of 20:1 (FeS to Cy5.5-NHS), and the mixture was stirred in an ice bath for 30 minutes. After stirring was completed, unbound Cy5.5-NHS was removed by centrifugal washing. Finally, the FeS nanosheets labeled with Cy5.5 are redispersed in PBS to prepare for subsequent modification steps.

Cell Uptake

RAW264.7 cells were seeded in laser confocal culture dishes at a density of 1×10^5 cells per dish, followed by the addition of FPRM-Cy5.5 at different concentrations and different treatments. The LIFU parameters were set at 1.0 MHz, 3 W/cm^2 for 60 s. After 4 h of incubation, the cells were stained with Hoechst 33342 for 15 minutes. Finally, intracellular fluorescent signals were imaged using the CLSM. Meanwhile, RAW cells were seeded in 6-well plates at a density of 2×10^5 cells per well and subjected to the same treatments. After collecting the cells, they were analyzed using a flow cytometer.

For the HUVEC cells uptake assay, cells were maintained in DMEM supplemented with 10 % FBS. Upon reaching confluence, HUVEC cells were activated by stimulation with LPS (1 μg/mL) for 24 h. Subsequently, non-activated or activated HUVEC cells were incubated with FPR-Cy5.5 or FPRM-Cy5.5. Afterward, the cells were stained with Hoechst 33342 and imaged by the CLSM. To investigate the importance of VCAM-1 on the interaction and cellular uptake of RMM-coated nanosheets, activated HUVEC cells were treated with 300 μg/mL VCAM-1 antibodies for 1 h before incubation with RMM-coated nanosheets. Then, after incubation with 100 μg of FPRM-Cy5.5 for 4 h, the cells were stained with Hoechst for visualization by CLSM.

Free-Radical-Scavenging Capacity of FPRM

To evaluate the free-radical-scavenging capacity of FPRM, typical antioxidant activity assays, including the DPPH• assay, PTIO assay, and •OH scavenging assay, were employed and monitored by UV−vis spectroscopy. For DPPH•, 0.04 mg mL^{-1} DPPH in ethanol was mixed with an equal volume of serial FPRM solutions, reacted in the dark for 30 min and then measured at 519 nm. For PTIO, a 0.05 mg mL^{-1} PTIO solution in PBS was prepared, combined 1:1 (v/v) with FPRM and incubated at room temperature for 2 h before recording absorbance at 557 nm. The •OH assay was performed according to the kit protocol, and •OH levels were quantified at 550 nm. All samples were centrifuged at 10000 rpm for 10 min to remove particulates prior to spectroscopic analysis. Each assay was performed in triplicate. The scavenging efficiency (%) was calculated as $[(A_0 - A)/A_0] \times 100$, where A_0 and A are the absorbances in the absence and presence of FPRM, respectively.

In vitro Inflammatory Cytokine Inhibition Experiment

RAW264.7 cells were seeded in culture plates and stimulated with LPS (1 μg/mL) for 24 h. Then, the cells were subjected to the following treatments: PBS, FRM, FPRM, FPRM + NIR, and FPRM + NIR + LIFU. The NIR parameters were set at 808 nm, 1.0 W/cm^2 for 5 min and the LIFU parameters were set at 1.0 MHz, 3 W/cm^2 for 60 s. After treatment, the cell culture medium was collected and centrifuged at 3000 rpm at 4°C for 10 minutes. ELISA kits were used to determine the levels of tumor necrosis factor (TNF-α), interleukin-6 (IL-6), and interleukin-1β (IL-1β) in the supernatant, respectively.

Western Blot

RAW264.7 cells were seeded into 6-well plates and stimulated with LPS (1 μg/mL) or ox-LDL (50 μg/ml) for 24 h. The cells were then treated as follows: PBS, FRM, FPRM, FPRM + NIR, and FPRM + NIR + LIFU. The NIR parameters were set at 808 nm, 1.0 W/cm^2 for 5 min and the LIFU parameters were set at 1.0 MHz, 3 W/cm^2 for 60 s. Subsequently, the cells were collected and lysed using a lysis buffer containing protease inhibitors. Western blot (WB) analysis was performed to detect the levels of iNOS, pAMPK/AMPK, LAMP1, LC3B-II/I, P62, ABCA1.

Establishment of a Combined Atherosclerosis and Hepatic Steatosis Mouse Model

Male $ApoE^{-/-}$ mice (8 weeks old) were fed a High Fat Diet (HFD) (21% fat and 0.15% cholesterol) for 12 weeks to establish a combined atherosclerosis/hepatic steatosis model. In this study, subsequent vascular and hepatic evaluations were performed in the same batch of model mice. After model establishment, aortic ultrasound imaging, as well as grayscale liver ultrasound and shear-wave elastography, were used to validate the model.

Drug distribution in vivo

To evaluate the in vivo biodistribution of FPRM, the atherosclerotic mice were randomly divided into two groups and intravenously injected with Cy5.5 fluorescently labeled FPR or FPRM (8 mg/kg). The mice were euthanized after injection for 0, 1, 1.5, 2, 4 h respectively. The aortas were collected for fluorescence imaging via the Small Animal Live Imaging System (IVIS®Lumina Series III, PerkinElmer, USA) and quantitative analysis. Meanwhile, the major organs (heart, liver, spleen, lung, and kidney) were collected from mice euthanized 4 h after injection for fluorescence imaging.

To further investigate whether LIFU could enhance the accumulation and retention of FPRM in the atherosclerotic plaque region, additional atherosclerotic mice were intravenously injected with Cy5.5-labeled FPRM (8 mg/kg) and randomly assigned to the FPRM group or the FPRM + LIFU group (*n* = 3 mice per group). At 1 h post-injection, mice in the FPRM + LIFU group were subjected to LIFU treatment (1.0 MHz, 3 W/cm^2, 60 s), whereas mice in the FPRM group received no ultrasound exposure. Then, the mice were euthanized at 2 h post-injection. The aortas were collected for fluorescence imaging via the Small Animal Live Imaging System (IVIS®Lumina Series III, PerkinElmer, USA) and quantitative analysis.

In vitro and in vivo MR Imaging

RAW264.7 cells were seeded in 6-well plates at a density of 3×10^5 cells/well and stimulated with LPS (1 μg/mL) for 24 h. The cells were treated with FPRM (with Fe^{2+} concentrations of 0, 2, 4, 8, 16, 32 μg/ml) for 6 hours. The above cells were made into gels and imaged by 9.4T MR. To evaluate the imaging of FPRM on atherosclerotic plaques in vivo, the atherosclerotic mice were randomly divided into 2 groups and intravenously injected with FPR or FPRM (8 mg/kg). 9.4T magnetic resonance scans were performed before and after injection, respectively. The MRI effect of atherosclerotic plaques before and after injection was calculated.

In Vivo Thermal Imaging

To evaluate the in vivo thermal imaging capability of FPRM, atherosclerotic mice were selected. Mice in the experimental group received FPRM (8 mg/kg) via tail vein injection, while the control group received an equal volume of PBS. At 2 h post-injection, the mice were anesthetized and placed in the prone position for laser irradiation. An 808 nm laser with a power density of 1.0 W cm^{-2} was used to non-invasively irradiate the abdominal aortic plaque region from the dorsal side for 5 minutes. During irradiation, an infrared thermal imaging camera was used to record the temperature changes in the corresponding region in real time, and the temperature at the projected area of the plaque region was analyzed from the thermal images.

Biosafety assay.

Mouse blood was centrifuged and washed to obtain red blood cells. Subsequently, the red blood cells were treated with different methods and incubated at 37°C for 3 hours, followed by

centrifugation and photographing. Finally, the absorbance of the samples at 542 nm was measured using an enzyme - labeled instrument to calculate the hemolysis rate. Meanwhile, at the end of the treatment, blood samples were collected from the mice for biochemical analysis and routine blood tests. The major organs of the mice were collected and stained with hematoxylin - and - eosin (H&E) for histological analysis.

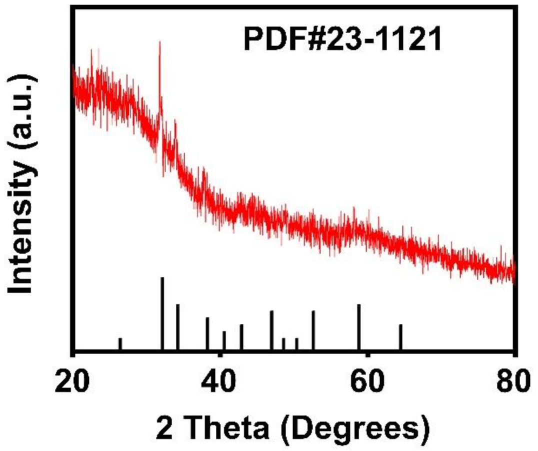

Fig. S1. XRD pattern of FeS nanosheets. The crystal structure measurements were performed on XRD (MiniFlex 600, Rigaku, Japan).

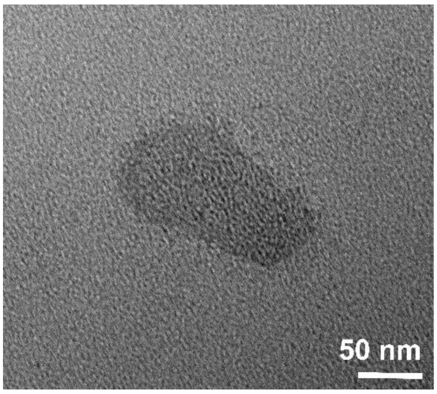

Fig. S2. TEM image of FPRM. RepresentativeTEM image of FPRM. Scale bar, 50 nm.

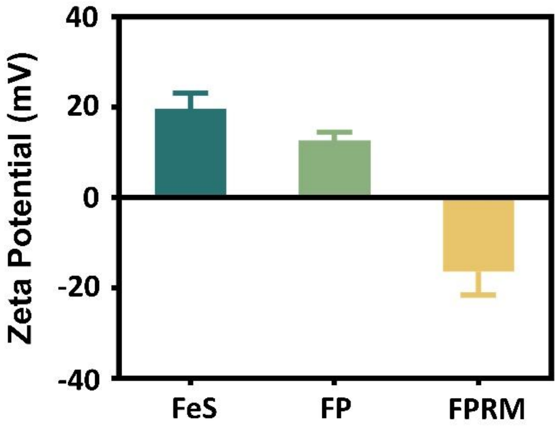

Fig. S3. ζ potential analysis of FeS, FP and FPRM. The particle size was measured using a DLS detector (Zetasizer Nano S90, Malvern, UK). Data were presented as means ± SD (n = 3 independently prepared samples).

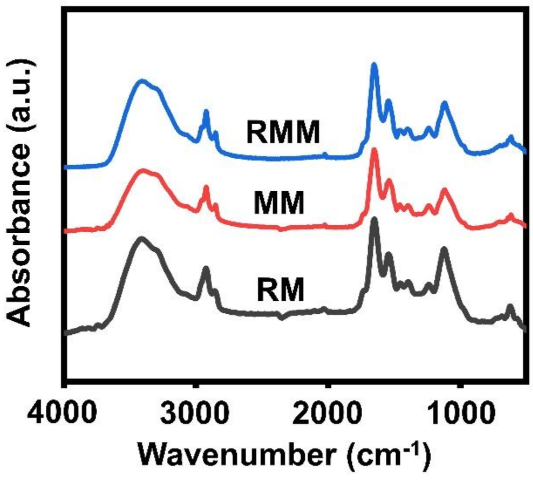

Fig. S4. FTIR of RM, MM and RMM. Fourier-transform infrared spectroscopy (FT-IR, Nicolet iS5, Thermo, USA) was employed to analyze RM, MM, and RMM.

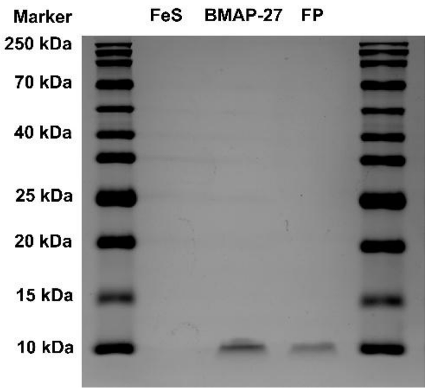

Fig. S5. Coomassie brilliant blue staining of SDS-PAGE. The grafting of BMAP-27 peptides onto FeS was evaluated by SDS-PAGE. The experiment was divided into three groups: the FeS group, the BMAP-27 peptides group, and the FP group. Images were captured using a gel imaging system (Biorad ChemiDoc MP, Bio-Rad, USA).

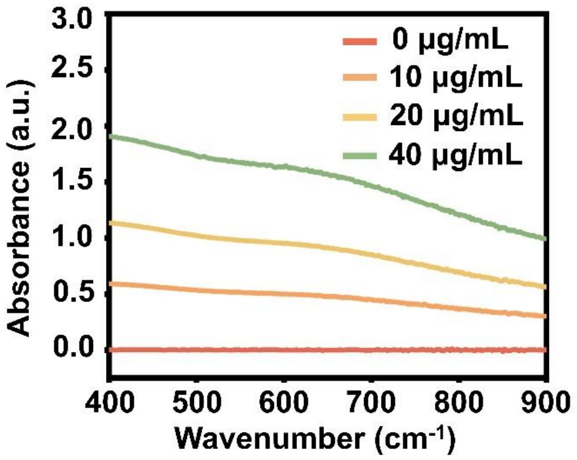

Fig. S6. UV-vis absorption spectra of FeS nanosheets at different concentrations. UV-vis absorption spectra of FeS nanosheets at 0, 10, 20, and 40 μg/mL.

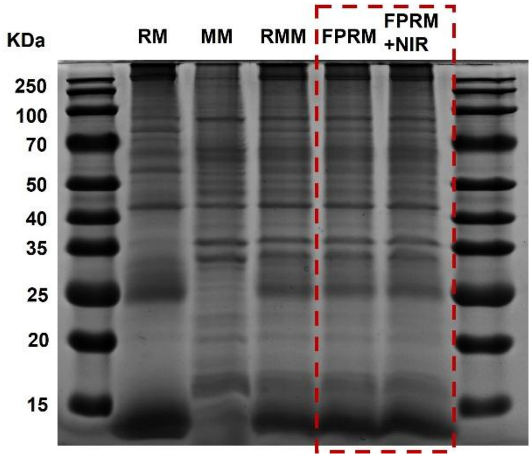

Fig. S7 SDS-PAGE analysis of membrane protein stability after NIR irradiation of FPRM. Protein profiles of red blood cell membrane (RM), macrophage membrane (MM), hybrid membrane (RMM), and membrane-integrated FPRM before and after NIR irradiation (808 nm, 1.0 W cm^{-2}, 5 min) were analyzed by Coomassie brilliant blue-stained SDS-PAGE.

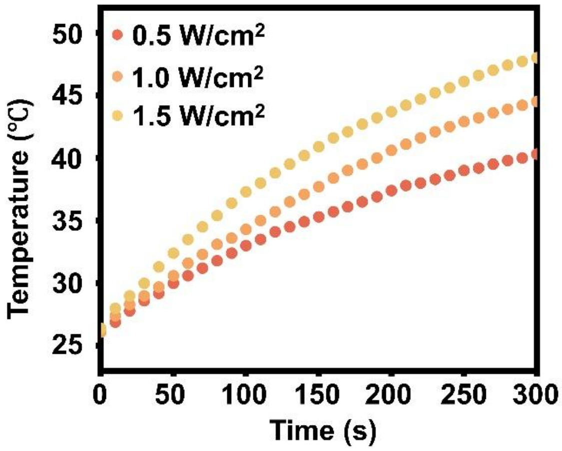

Fig. S8. Photothermal heating curves of FPRM under various irradiation power densities. Temperature profiles of FPRM at a concentration of 10 μg/mL under 808 nm laser irradiation at power densities of 0.5, 1.0, and 1.5 W/cm².

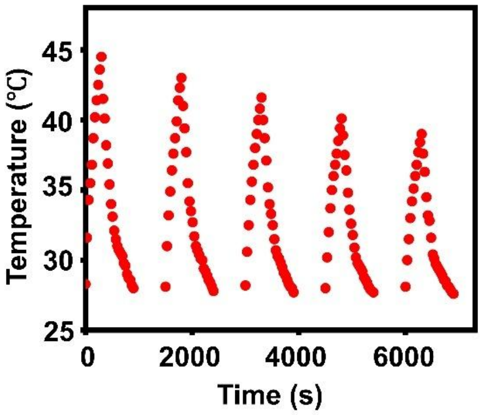

Fig. S9. Photothermal cycling stability of FPRM under repeated NIR irradiation. An aqueous dispersion of FPRM (10 μg/mL) was exposed to five cycles of 808 nm laser irradiation at 1.0 W/cm². In each cycle, the dispersion was irradiated for 5 min and then naturally cooled to room temperature, while the temperature was recorded.

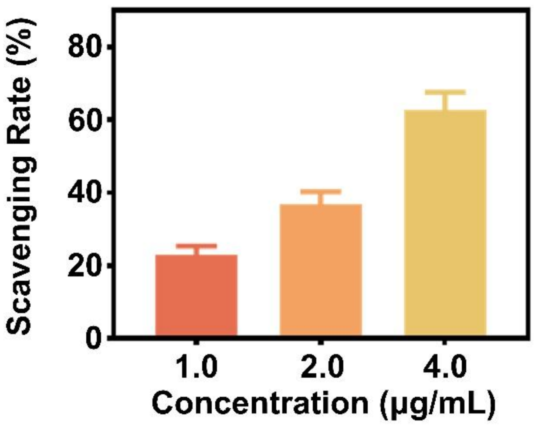

Fig. S10. Quantitative analysis of the DPPH•-scavenging capacity of FPRM. The DPPH•-scavenging efficiency of FPRM at different concentrations was quantified. The scavenging efficiency (%) was calculated as $[(A_0 - A)/A_0] \times 100$, where A_0 and A represent the absorbances in the absence and presence of FPRM, respectively. Data were presented as means ± SD (*n* = 3 independently prepared samples).

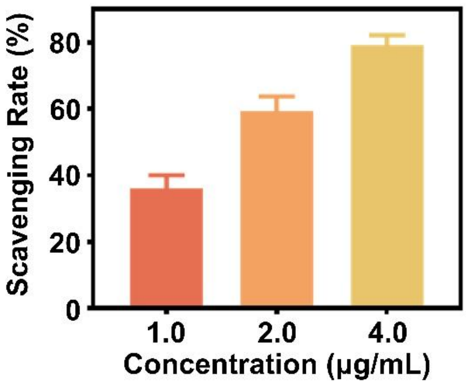

Fig. S11. Quantitative analysis of the PTIO•-scavenging capacity of FPRM. PTIO•-scavenging efficiency of FPRM at different concentrations. The scavenging efficiency (%) was calculated as $[(A_0 - A)/A_0] \times 100$, where A_0 and A are the absorbances in the absence and presence of FPRM, respectively. Data were presented as means ± SD (*n* = 3 independently prepared samples).

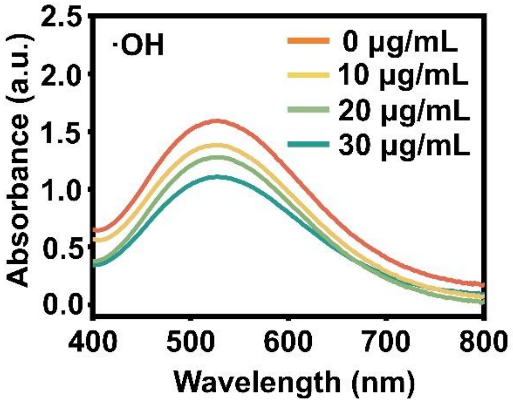

Fig. S12. UV-vis analysis of the ·OH-eliminating capability of FPRM. The ·OH-eliminating capacity of FPRM at different concentrations was evaluated by UV-vis spectroscopy. The ·OH assay was performed according to the manufacturer's protocol, and the ·OH level was quantified at 550 nm. All samples were centrifuged at 10000 rpm for 10 min to remove particulates prior to spectroscopic analysis.

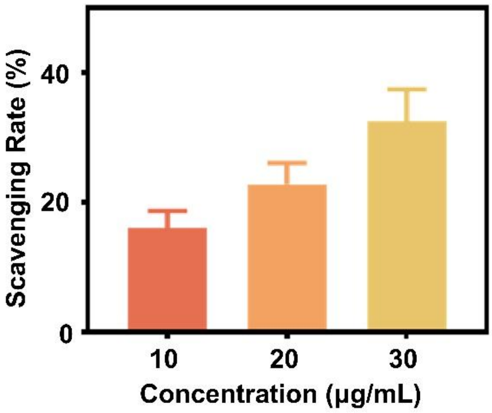

Fig. S13. Quantitative analysis of the ·OH-scavenging capacity of FPRM. ·OH-scavenging efficiency of FPRM at different concentrations. The scavenging efficiency (%) was calculated as $[(A_0 - A)/A_0] \times 100$, where A_0 and A are the absorbances in the absence and presence of FPRM, respectively. Data were presented as means ± SD (*n* = 3 independently prepared samples).

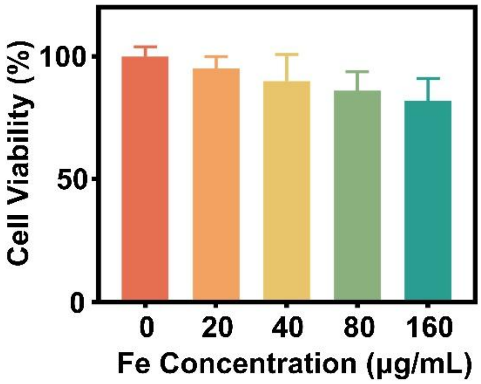

Fig. S14. Cell viability of HUVECs after incubation with FPRM. HUVECs were incubated with FPRM at different Fe concentrations (0, 20, 40, 80, and 160 μg/mL) for 24 h, and cell viability was determined by the MTT assay. Data were presented as means ± SD (*n* = 6 technical replicates).

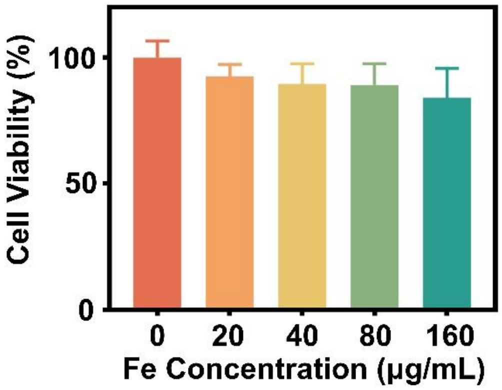

Fig. S15. Cell viability of RAW 264.7 cells after incubation with FPRM. RAW 264.7 cells were incubated with FPRM at different Fe concentrations (0, 20, 40, 80, and 160 μg/mL) for 24 h, and cell viability was determined by the MTT assay. Data were presented as means ± SD (*n* = 6 technical replicates).

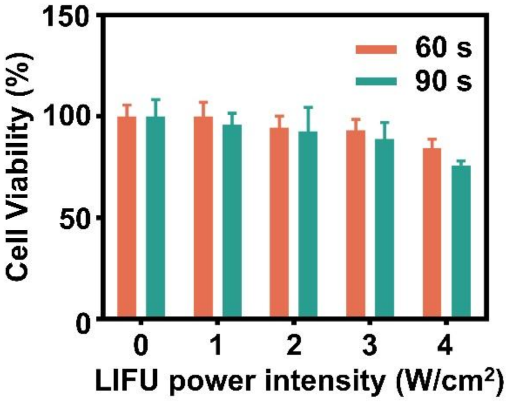

Fig. S16. Cell viability of RAW 264.7 cells after ultrasound treatment at different power intensities and exposure durations. RAW 264.7 cells were exposed to ultrasound at a fixed frequency of 1 MHz with different power intensities (0, 1, 2, 3, and 4 W/cm²) for 60 or 90 s, and cell viability was determined by the MTT assay. Data were presented as means ± SD (*n* = 4 technical replicates).

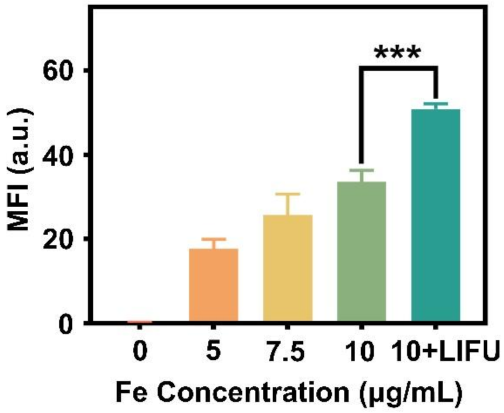

Fig. S17. Quantitative analysis of cellular uptake under different treatment conditions. Mean fluorescence intensity (MFI) was used to quantify the cellular uptake after different treatments. Statistical comparisons were conducted using one-way ANOVA. ***P < 0.001. Data were presented as means ± SD (n = 3 independent experiments).

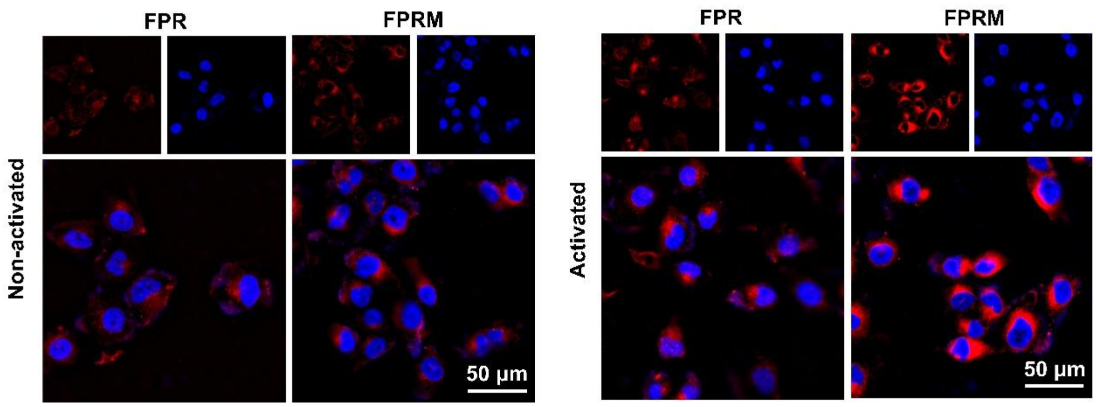

Fig. S18. CLSM images of cellular uptake of FPR and FPRM in non-activated and activated HUVECs. Non-activated or LPS-activated HUVECs (1 μg/mL, 24 h) were incubated with FPR-Cy5.5 or FPRM-Cy5.5, followed by Hoechst 33342 staining and CLSM imaging. Scale bar, 50 μm.

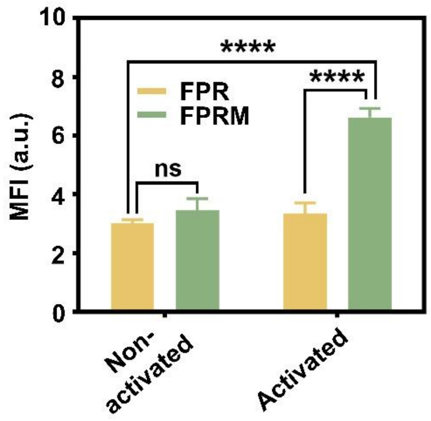

Fig. S19. Quantitative analysis of the uptake of FPR and FPRM by non-activated and activated HUVECs cells. The uptake of FPR and FPRM by non-activated and activated HUVECs cells was quantified by MFI. Statistical comparisons were conducted using one-way ANOVA. ****$P < 0.0001$. Data were presented as means ± SD ($n = 3$ independent experiments).

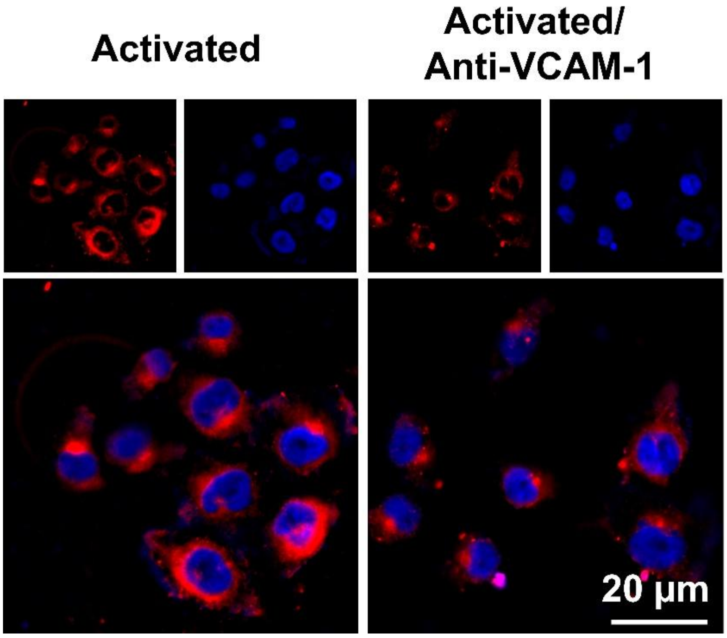

Fig. S20. CLSM images of cellular uptake of FPRM in activated HUVECs with or without anti-VCAM-1 pretreatment. Activated HUVECs were pretreated with anti-VCAM-1 antibody (300 μg/mL) for 1 h and then incubated with FPRM-Cy5.5 for 4 h. After Hoechst 33342 staining, the cells were imaged by CLSM. Scale bar, 20 μm.

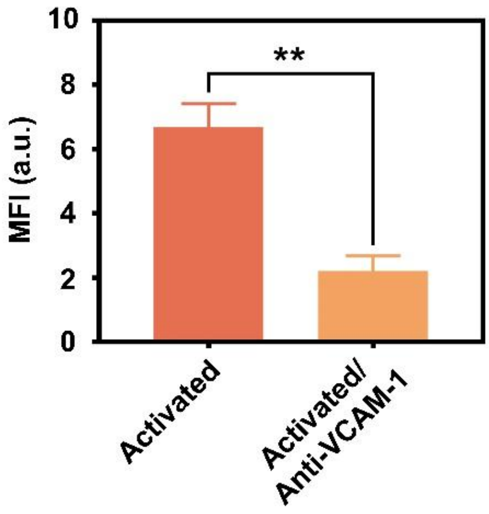

Fig. S21. Quantitative analysis of the cellular uptake of FPRM in activated HUVECs with or without anti-VCAM-1 pretreatment. The uptake of FPRM in activated HUVECs with or without anti-VCAM-1 pretreatment was quantified by MFI. Statistical comparisons were conducted using one-way ANOVA. **P < 0.01. Data were presented as means ± SD (n = 3 independent experiments).

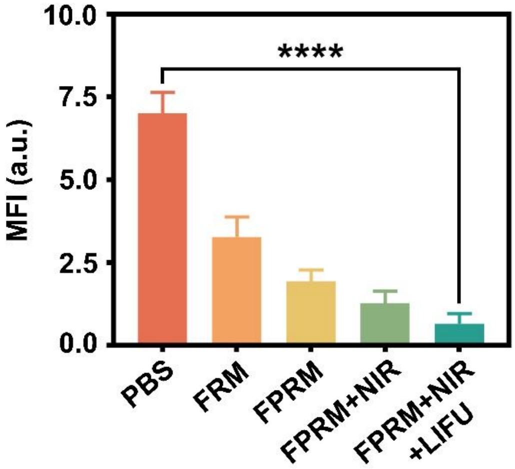

Fig. S22. Quantitative analysis of intracellular ROS levels in M1 macrophages after different treatments. Intracellular ROS levels in M1 macrophages were quantified by MFI using DCFH-DA as the indicator after the indicated treatments. Statistical comparisons were conducted using one-way ANOVA. ****P < 0.0001. Data were presented as means ± SD (n = 3 independent experiments).

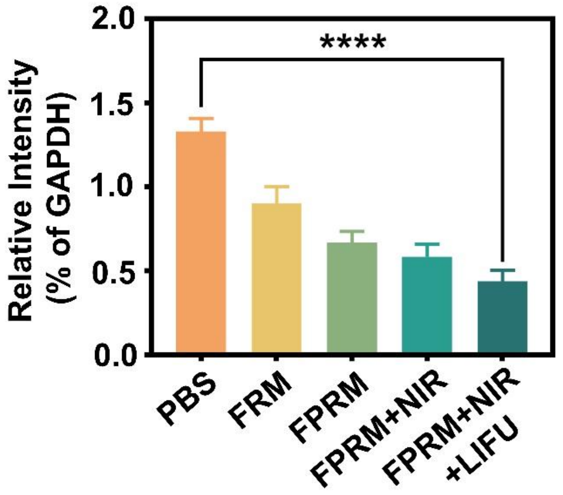

Fig. S23. Quantitative analysis of iNOS expression in M1 macrophages after different treatments. The relative iNOS expression in M1 macrophages after the indicated treatments was quantified and normalized to GAPDH. Statistical significance was analyzed by one-way ANOVA. ****P < 0.0001. Data were presented as means ± SD (n = 3 independent experiments).

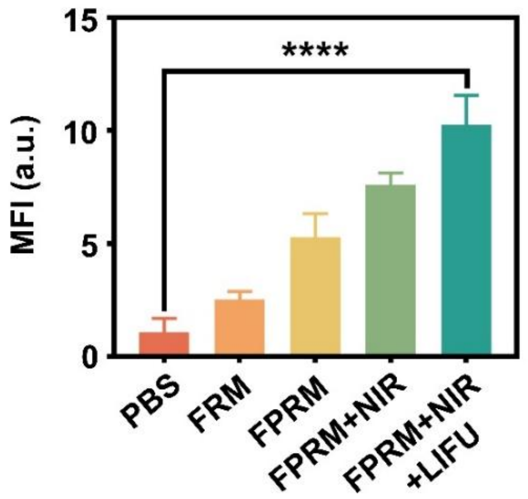

Fig. S24. CD206 expression in LPS-prestimulated RAW 264.7 cells was quantified by MFI after the indicated treatments. Statistical comparisons were conducted using one-way ANOVA. ****P < 0.0001. Data were presented as means ± SD (n = 3 independent experiments).

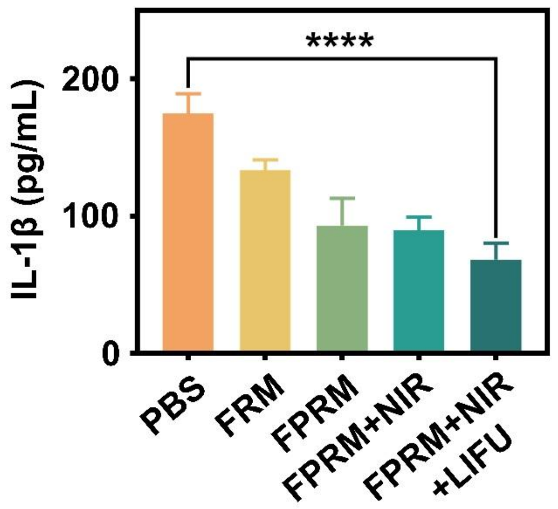

Fig. S25. IL-1β levels in LPS-stimulated RAW 264.7 cells after different treatments. The level of the inflammatory cytokine IL-1β in LPS-stimulated RAW 264.7 cells was measured after the indicated treatments. Statistical significance was analyzed by one-way ANOVA. ****$P <$ 0.0001. Data were presented as means ± SD (n = 3 independent experiments).

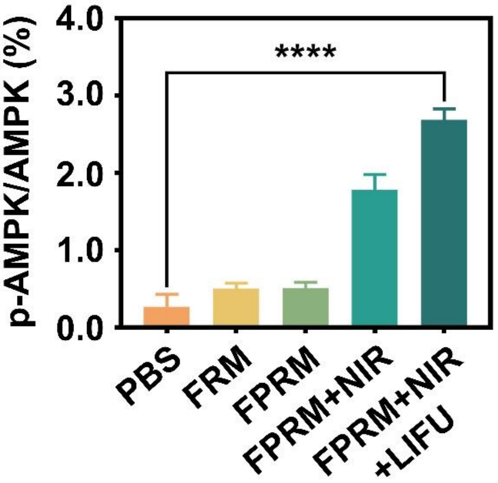

Fig. S26. Quantitative analysis of the p-AMPK/AMPK ratio in foam cells after different treatments. The relative p-AMPK/AMPK ratio in foam cells after the indicated treatments was quantified. Statistical comparisons were conducted using one-way ANOVA. ****P < 0.0001. Data were presented as means ± SD (n = 3 independent experiments).

.

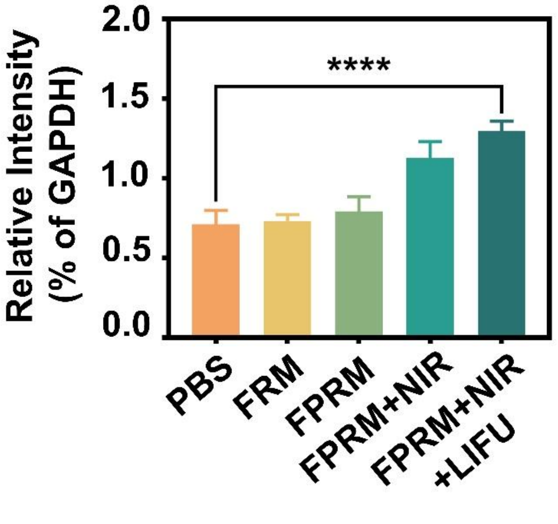

Fig. S27. Quantitative analysis of LAMP1 expression in foam cells after different treatments. The relative LAMP1 expression in foam cells after the indicated treatments was quantified and normalized to GAPDH. Statistical comparisons were conducted using one-way ANOVA. ****$P < 0.0001$. Data were presented as means ± SD ($n = 3$ independent experiments).

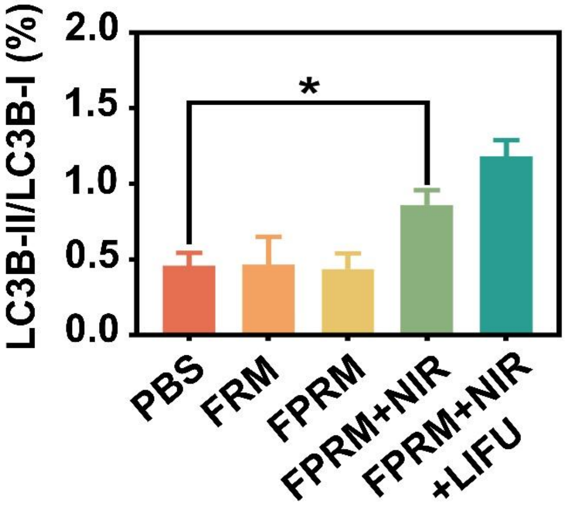

Fig. S28. Quantitative analysis of the LC3-II/LC3-I ratio in foam cells after different treatments. The LC3-II/LC3-I ratio in foam cells after the indicated treatments was quantified. Statistical comparisons were conducted using one-way ANOVA. *$P < 0.05$. Data were presented as means ± SD ($n = 3$ independent experiments).

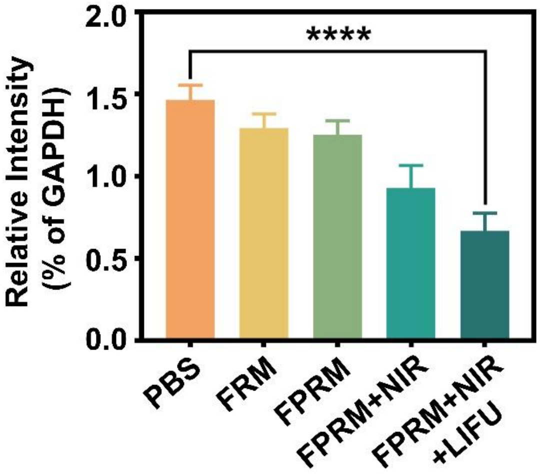

Fig. S29. Quantitative analysis of p62 expression in foam cells after different treatments. The relative p62 expression in foam cells after the indicated treatments was quantified and normalized to GAPDH. Statistical comparisons were conducted using one-way ANOVA. $^{****}P < 0.0001$. Data were presented as means ± SD (n = 3 independent experiments).

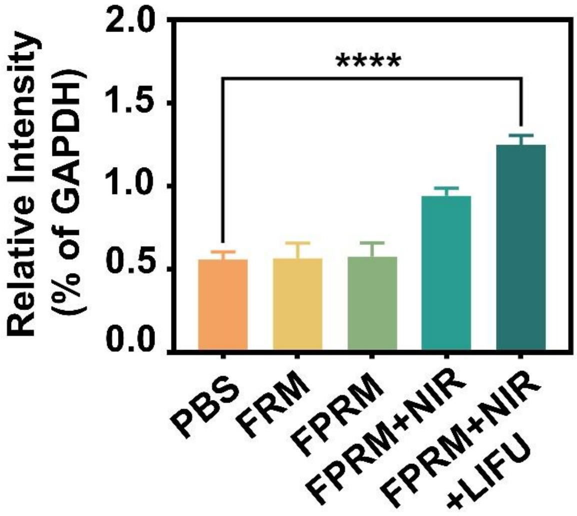

Fig. S30. Quantitative analysis of ABCA1 expression in foam cells after different treatments. The relative ABCA1 expression in foam cells after the indicated treatments was quantified and normalized to GAPDH. Statistical comparisons were conducted using one-way ANOVA. ****$P < 0.0001$. Data were presented as means ± SD ($n = 3$ independent experiments).

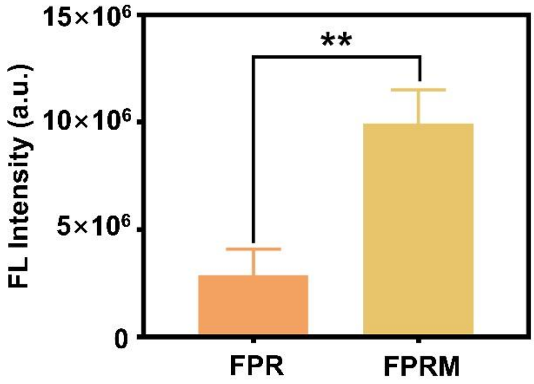

Fig. S31. Quantitative fluorescence intensity analysis of aortas from atherosclerotic mice after intravenous administration of Cy5.5-labeled FPR or FPRM. Fluorescence intensity of aortas from atherosclerotic mice was quantified at 2 h after intravenous injection of Cy5.5-labeled FPR or Cy5.5-labeled FPRM. Statistical comparisons were conducted using one-way ANOVA. $^{**}P < 0.01$. Data were presented as means ± SD (n = 3 biologically independent mice).

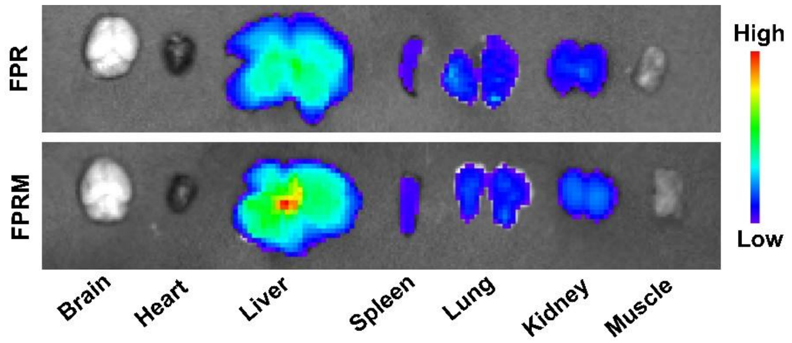

Fig. S32. Ex vivo fluorescence imaging of isolated organs from atherosclerotic mice after intravenous administration of Cy5.5-labeled FPR or FPRM. Atherosclerotic mice were intravenously injected with Cy5.5-labeled FPR or FPRM (8 mg/kg). At 4 h post-injection, the brain, heart, liver, spleen, lung, kidney, and muscle were collected for ex vivo fluorescence imaging using an IVIS imaging system (IVIS Lumina Series III, PerkinElmer, USA).

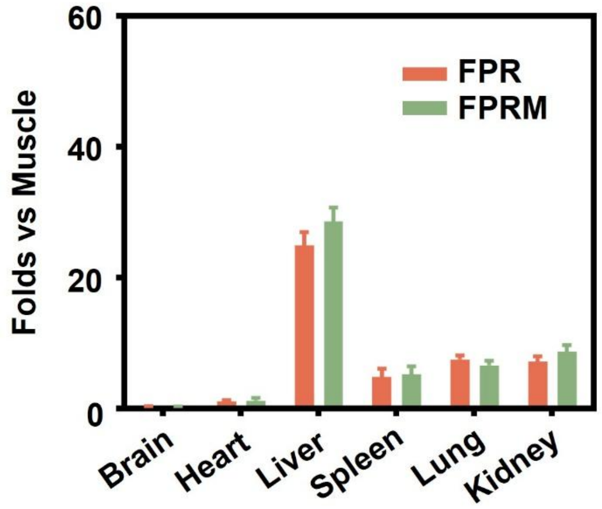

Fig. S33. Quantitative ex vivo fluorescence analysis of major organs from atherosclerotic mice after intravenous administration of Cy5.5-labeled FPR or FPRM. Fluorescence intensity of the brain, heart, liver, spleen, lung, and kidney was quantified at 4 h after intravenous injection of Cy5.5-labeled FPR or Cy5.5-labeled FPRM and normalized to muscle. Data are presented as means ± SD (*n* = 3 biologically independent mice).

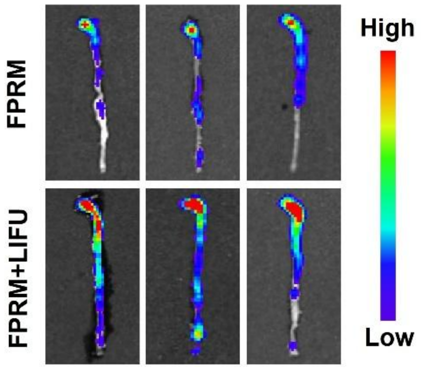

Fig. S34 Ex vivo fluorescence images of aortas after different treatments. Representative ex vivo fluorescence images of aortas collected from mice treated with FPRM or FPRM + LIFU.

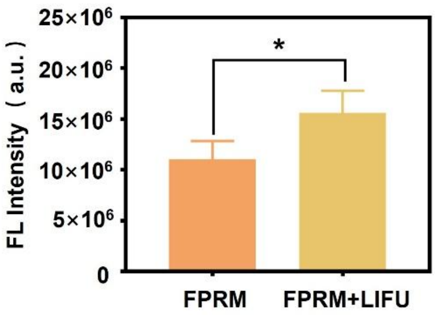

Fig. S35. Quantitative fluorescence intensity analysis of aortas from atherosclerotic mice after intravenous administration of Cy5.5-labeled FPRM with or without LIFU treatment. Fluorescence intensity of aortas from atherosclerotic mice was quantified at 2 h after intravenous injection of Cy5.5-labeled FPRM or Cy5.5-labeled FPRM followed by LIFU treatment. Statistical comparisons were conducted using one-way ANOVA. $^*P < 0.05$. Data were presented as means ± SD (n = 3 biologically independent mice).

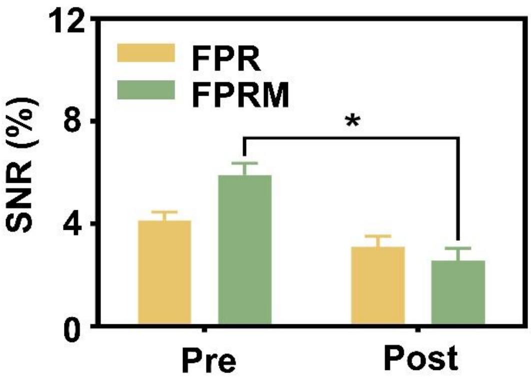

Fig. S36. SNR in the plaque region of atherosclerotic mice before and after intravenous administration of FPR or FPRM. Statistical comparisons were conducted using one-way ANOVA. $^*P < 0.05$. Data were presented as means ± SD.

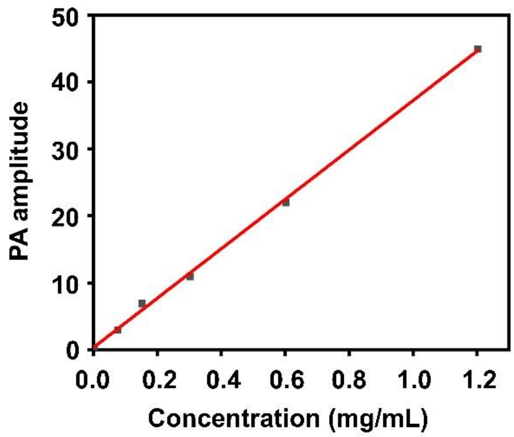

Fig. S37. Photoacoustic signal intensity of FPRM at different concentrations. Photoacoustic imaging (PAI) was performed to evaluate the signal intensity of FPRM at different concentrations, and the corresponding PA amplitudes were shown.

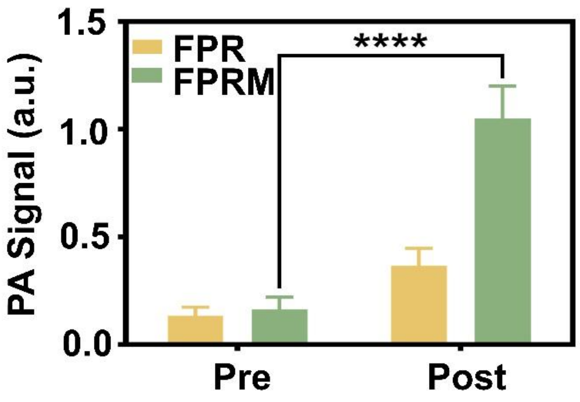

Fig. S38. Quantitative analysis of PA intensity in the plaque region pre- and post-injection of FPR or FPRM. Statistical comparisons were conducted using one-way ANOVA. ****P < 0.0001. Data were presented as means ± SD.

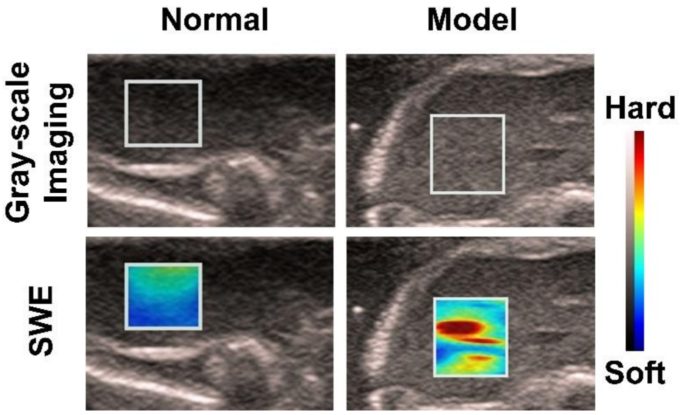

Fig. S39. Ultrasound validation of hepatic steatosis in the atherosclerosis/hepatic steatosis comorbidity model. Representative gray-scale ultrasound images and shear wave elastography (SWE) images of the liver in normal mice and ApoE^{-}/$^{-}$ model mice after 12 weeks of high-fat diet feeding.

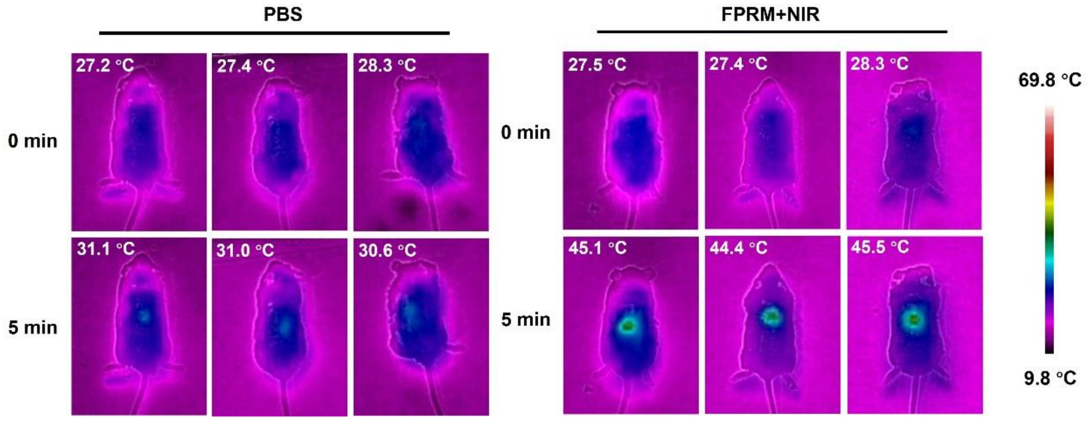

Fig. S40. In vivo infrared thermography of local mild photothermal heating under 808-nm irradiation. Representative infrared thermal images of atherosclerotic mice recorded at 0 min and after 5 min of 808-nm laser irradiation (1.0 W cm^{-2}) following intravenous administration of PBS or FPRM.

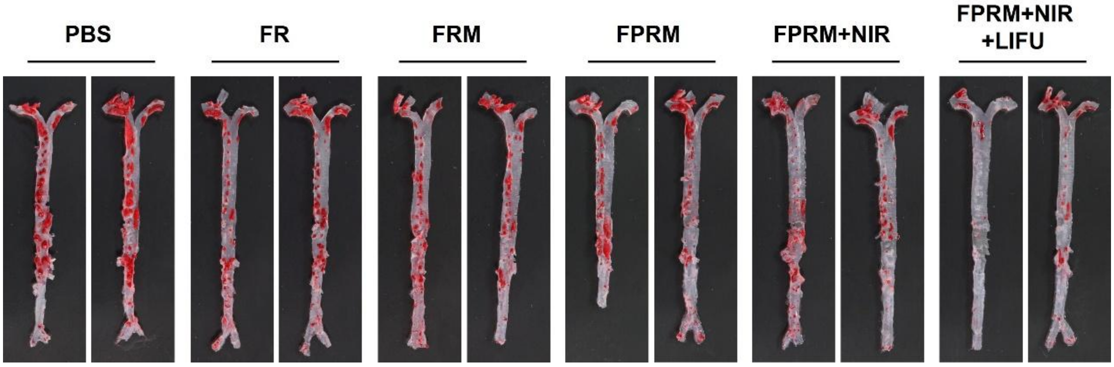

Fig. S41. Representative en face Oil Red O staining images of aortas from atherosclerotic mice after different treatments. Representative en face Oil Red O staining images of whole aortas harvested from atherosclerotic mice after treatment with PBS, FR, FRM, FPRM, FPRM + NIR, or FPRM + NIR + LIFU.

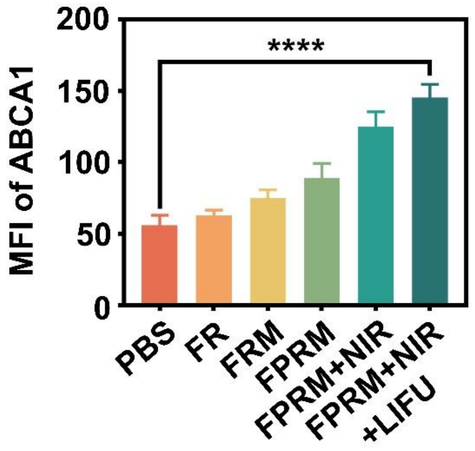

Fig. S42. Quantitative analysis of ABCA1 expression in plaques after different treatments. ABCA1 expression in plaques after the indicated treatments was quantified by mean fluorescence intensity (MFI). Statistical comparisons were conducted using one-way ANOVA. ****P < 0.0001. Data were presented as means ± SD (n = 3 biologically independent mice).

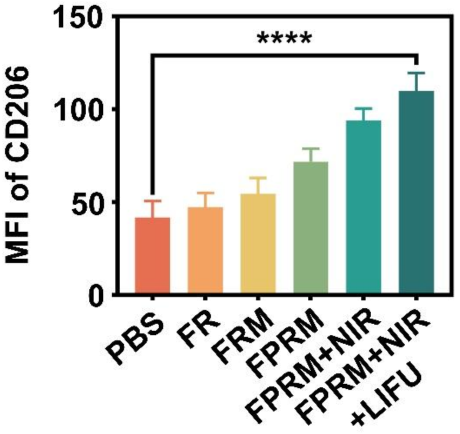

Fig. S43. Quantitative analysis of CD206 expression in atherosclerotic plaques after different treatments. CD206 expression in atherosclerotic plaques after the indicated treatments was quantified by MFI. Statistical comparisons were conducted using one-way ANOVA. ****$P < 0.0001$. Data were presented as means ± SD ($n = 3$ biologically independent mice).

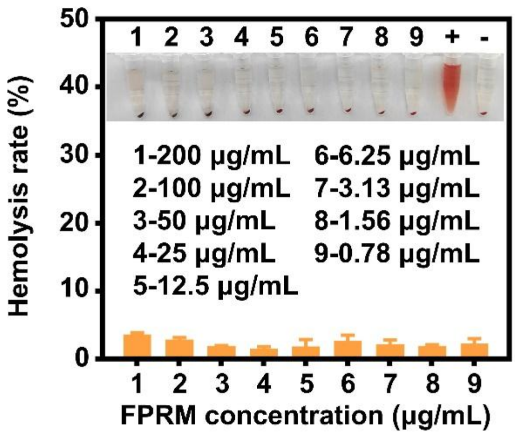

Fig. S44. Hemolysis images and hemolysis rates of FPRM at different concentrations. Red blood cells isolated from mouse blood were incubated with FPRM at different concentrations at 37°C for 3 h. After centrifugation, representative hemolysis images were recorded, and the absorbance of the supernatants at 542 nm was measured to calculate the hemolysis rate. Data were presented as means ± SD (*n* = 3 technical replicates).

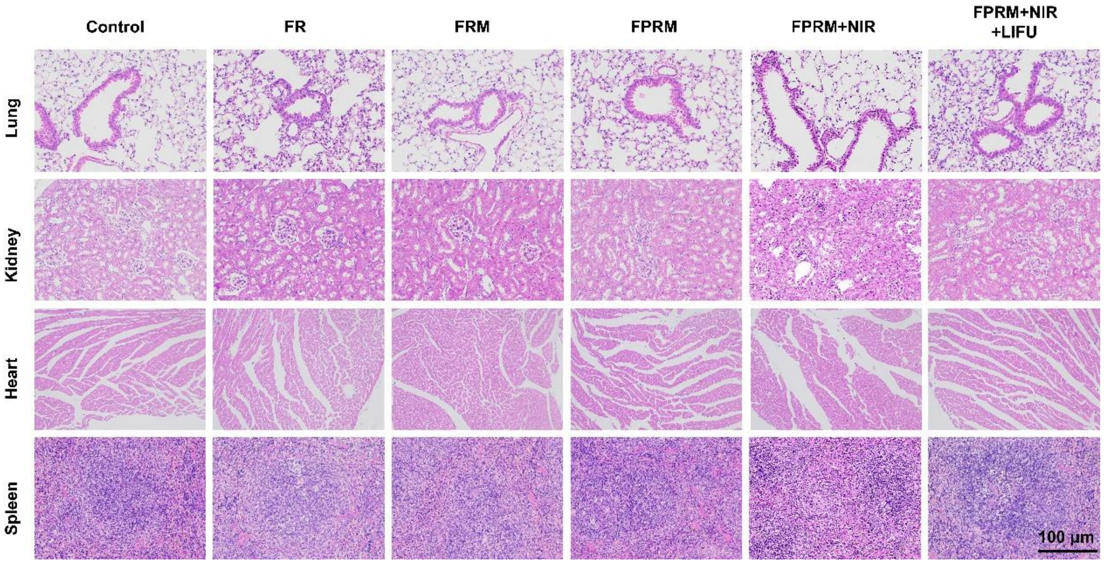

Fig. S45. H&E staining of major organs after different treatments. Representative H&E-stained sections of the lung, kidney, heart, and spleen collected after the indicated treatments. Scale bar, 100 μm.

REFERENCES

1. H. Xu, P. She, Z. Zhao, B. Ma, G. Li, Y. Wang, Duplex desponsive nanoplatform with cascade targeting for atherosclerosis photoacoustic diagnosis and multichannel combination therapy. *Adv. Mater.* **35**, e2300439 (2023).

2. M. Bekbossynova, T. Saliev, T. Ivanova-Razumova, S. Andossova, A. Kali, G. Myrzakhmetova, Beyond cholesterol: Emerging risk factors in atherosclerosis. *J. Clin. Med.* **14**, 2352 (2025).

3. J. Sun, P. Singh, A. Shami, E. Kluza, M. Pan, D. Djordjevic, N. B. Michaelsen, C. Kennbäck, N. N. van der Wel, M. Orho-Melander, J. Nilsson, I. Formentini, K. Conde-Knape, E. Lutgens, A. Edsfeldt, I. Gonçalves, Spatial transcriptional mapping reveals site-specific pathways underlying human atherosclerotic plaque rupture. *J. Am. Coll. Cardiol.* **81**, 2213–2227 (2023).

4. A. Ajoolabady, D. Pratico, L. Lin, C. S. Mantzoros, S. Bahijri, J. Tuomilehto, J. Ren, Inflammation in atherosclerosis: Pathophysiology and mechanisms. *Cell Death Dis.* **15**, 817 (2024).

5. S. Miki, M. Takashima, J.-I. Suzuki, Anti-atherosclerotic effect of aged garlic extract: Mode of action and therapeutic benefits. *Exp. Ther. Med.* **29**, 104 (2025).

6. Y. Yu, S. Liu, L. Yang, P. Song, Z. Liu, X. Liu, X. Yan, Q. Dong, Roles of reactive oxygen species in inflammation and cancer. *MedComm* **5**, e519 (2024).

7. M. Yu, S. Wang, D. Lin, Mechanism and application of biomaterials targeting reactive oxygen species and macrophages in inflammation. *Int. J. Mol. Sci.* **26**, 245 (2025).

8. M. Hunt, M. Torres, E. Bachar-Wikstrom, J. D. Wikstrom, Cellular and molecular roles of reactive oxygen species in wound healing. *Commun. Biol.* **7**, 1534 (2024).

9. P. Xiang, V. Blanchard, G. A. Francis, Smooth muscle cell-macrophage interactions leading to foam cell formation in atherosclerosis: Location, location, location. *Front. Physiol.* **13**, 921597 (2022).

10. G. He, Y. Ni, R. Hua, H. Wan, Y. Tan, Q. Chen, S. Xu, Y. Yang, L. Zhang, W. Shu, K.-B. Huang, Y. Mo, H. Liang, M. Chen, Latexin deficiency limits foam cell formation and ameliorates atherosclerosis by promoting macrophage phenotype differentiation. *Cell Death Dis.* **15**, 754 (2024).

11. Z. Ouyang, J. Zhong, J. Shen, Y. Zeng, The cell origins of foam cell and lipid metabolism regulated by mechanical stress in atherosclerosis. *Front. Physiol.* **14**, 1179828 (2023).

12. D. Kapuria, V. K. Takyar, O. Etzion, P. Surana, J. H. O'Keefe, C. Koh, Association of hepatic steatosis with subclinical atherosclerosis: Systematic review and meta-analysis. *Hepatol. Commun.* **2**, 873–883 (2018).

13. B. Zhu, H. Wu, K. S. Li, S. Eisa-Beygi, B. Singh, D. R. Bielenberg, W. Huang, H. Chen, Two sides of the same coin: Non-alcoholic fatty liver disease and atherosclerosis. *Vascul. Pharmacol.* **154**, 107249 (2024).

14. E. Björnson, D. Samaras, M. Levin, F. Bäckhed, G. Bergström, A. Gummesson, The impact of steatotic liver disease on coronary artery disease through changes in the plasma lipidome. *Sci. Rep.* **14**, 22307 (2024).

15. F. Baratta, L. D'Erasmo, S. Bini, D. Pastori, F. Angelico, M. Del Ben, M. Arca, A. Di Costanzo, Heterogeneity of non-alcoholic fatty liver disease (NAFLD): Implication for cardiovascular risk stratification. *Atherosclerosis* **357**, 51–59 (2022).

16. Y.-Z. Huang, J.-X. Ma, Y.-J. Bian, Q.-R. Bai, Y.-H. Gao, S.-K. Di, Y.-T. Lei, H. Yang, X.-N. Yang, C.-Y. Shao, W.-H. Wang, P. Cao, C.-Z. Li, M. X. Zhu, M.-Y. Sun, Y. Yu, TRPV1 analgesics disturb core body temperature via a biased allosteric mechanism involving conformations distinct from that for nociception. *Neuron* **112**, 1815–1831.e4 (2024).

17. R. Wang, J. Jiang, X. Zhou, Z. Wan, P. Zhang, S. Wang, Rapid regulation of local temperature and transient receptor potential vanilloid 1 ion channels with wide-field plasmonic thermal microscopy. *Anal. Chem.* **94**, 14503–14508 (2022).

18. H. Fang, L. Huang, F. Lv, B. Hu, H. Liu, Z. Huang, Y. Sun, W. Zhou, X. Wang, Dual-responsive targeted atherosclerosis therapy through a multi-effective nanoplatform with anti-inflammatory, lipid-regulating and autophagy. *Chem. Eng. J.* **454**, 140067 (2023).

19. X. Li, R. Zhu, H. Jiang, Q. Yin, J. Gu, J. Chen, X. Ji, X. Wu, H. Fu, H. Wang, X. Tang, Y. Gao, B. Wang, Y. Ji, H. Chen, Autophagy enhanced by curcumin ameliorates inflammation in atherogenesis via the TFEB-P300-BRD4 axis. *Acta Pharm. Sin. B* **12**, 2280–2299 (2022).

20. H. Xu, J. Duan, J. Tao, W. Wang, Y. Wu, S. Dai, J. Ren, Oxidized LDL regulates endothelin-1 and oxidative stress in vascular endothelial cells: Role of extracellular regulated kinase1/2 (ERK1/2). *Int. J. Drug Discov. Pharmacol.* **2**, 18–29 (2023).

21. F. Bonacina, X. Zhang, N. Manel, L. Yvan-Charvet, B. Razani, G. D. Norata, Lysosomes in the immunometabolic reprogramming of immune cells in atherosclerosis. *Nat. Rev. Cardiol.* **22**, 149–164 (2025).

22. Y. Duan, K. Gong, S. Xu, F. Zhang, X. Meng, J. Han, Regulation of cholesterol homeostasis in health and diseases: From mechanisms to targeted therapeutics. *Signal Transduct. Target. Ther.* **7**, 265 (2022).

23. L. G. B. Ferreira, J. V. Faria, J. P. S. dos Santos, R. X. Faria, Capsaicin: TRPV1-independent mechanisms and novel therapeutic possibilities. *Eur. J. Pharmacol.* **887**, 173356 (2020).

24. A. M. Chapa-Oliver, L. Mejía-Teniente, Capsaicin: From plants to a cancer-suppressing agent. *Molecules* **21,** 931 (2016).

25. Y. Wang, K. Zhang, T. Li, A. Maruf, X. Qin, L. Luo, Y. Zhong, J. Qiu, S. McGinty, G. Pontrelli, X. Liao, W. Wu, G. Wang, Macrophage membrane functionalized biomimetic nanoparticles for targeted anti-atherosclerosis applications. *Theranostics* **11**, 164–180 (2021).

26. D. Wang, H. Dong, M. Li, Y. Cao, F. Yang, K. Zhang, W. Dai, C. Wang, X. Zhang, Erythrocyte-cancer hybrid membrane camouflaged hollow copper sulfide nanoparticles for prolonged circulation life and homotypic-targeting photothermal/chemotherapy of melanoma. *ACS Nano* **12**, 5241–5252 (2018).
